# Control over single-cell distribution of G1 lengths by WNT governs pluripotency

**DOI:** 10.1371/journal.pbio.3000453

**Published:** 2019-09-26

**Authors:** Jiwon Jang, Dasol Han, Mahdi Golkaram, Morgane Audouard, Guojing Liu, Daniel Bridges, Stefan Hellander, Alex Chialastri, Siddharth S. Dey, Linda R. Petzold, Kenneth S. Kosik

**Affiliations:** 1 Department of Molecular, Cellular, and Developmental Biology, Neuroscience Research Institute, University of California, Santa Barbara, Santa Barbara, California, United States of America; 2 Department of Life Sciences, Pohang University of Science and Technology, Pohang, Republic of Korea; 3 Department of Mechanical Engineering, University of California, Santa Barbara, Santa Barbara, California, United States of America; 4 Department of Physics, University of California, Santa Barbara, Santa Barbara, California, United States of America; 5 Department of Computer Science, University of California, Santa Barbara, Santa Barbara, California, United States of America; 6 Department of Chemical Engineering, University of California, Santa Barbara, Santa Barbara, California, United States of America; 7 Center for Bioengineering, University of California Santa Barbara, Santa Barbara, California, United States of America; University of Copenhagen, DENMARK

## Abstract

The link between single-cell variation and population-level fate choices lacks a mechanistic explanation despite extensive observations of gene expression and epigenetic variation among individual cells. Here, we found that single human embryonic stem cells (hESCs) have different and biased differentiation potentials toward either neuroectoderm or mesendoderm depending on their G1 lengths before the onset of differentiation. Single-cell variation in G1 length operates in a dynamic equilibrium that establishes a G1 length probability distribution for a population of hESCs and predicts differentiation outcome toward neuroectoderm or mesendoderm lineages. Although sister stem cells generally share G1 lengths, a variable proportion of cells have asymmetric G1 lengths, which maintains the population dispersion. Environmental Wingless-INT (WNT) levels can control the G1 length distribution, apparently as a means of priming the fate of hESC populations once they undergo differentiation. As a downstream mechanism, global 5-hydroxymethylcytosine levels are regulated by G1 length and thereby link G1 length to differentiation outcomes of hESCs. Overall, our findings suggest that intrapopulation heterogeneity in G1 length underlies the pluripotent differentiation potential of stem cell populations.

## Abstract 2

The link between single-cell variation and population-level fate choices lacks a mechanistic explanation. This study finds that the duration of the G1 cell cycle phase in stem cells varies within the population, giving rise to a probability distribution of G1 length that is responsive to Wnt signalling and that predicts cells’ differentiation potential upon exit from pluripotency.

## Introduction

How a pluripotent population decides among fate choices is a highly relevant question of intense interest. Although growth conditions can deterministically direct the population-level outcome of stem cells once differentiation begins, the contribution of single-cell variation within the stem cell population [1–12] to the collective fate decision is poorly understood. In the absence of distinct cell types that establish stable variation across the population, stem cell variation arises from dynamic physiological events such as the cell cycle, changes in the microenvironment, and stochasticity that together establish a dynamic equilibrium. From this perspective, individual cells transit between distinct metastable states while maintaining the overall structure of the population [5,13–15]. Underlying stem cell heterogeneity is the influence of variations in the activity of signaling pathways at the single-cell level such as Wingless-INT (WNT), Bone morphogenetic protein (BMP), Nodal growth differentiation factor (NODAL), and Fibroblast growth factor (FGF) that can confer transient lineage biases to pluripotent stem cell subpopulations [16,17]. Highly variable gene expression patterns among single cells arise from the particularly permissive and dynamic chromatin structure of stem cells [18]. At the population level, well-defined robust behavior emerges from stochastic dynamics at the single-cell level [19].

Pluripotent stem cells have a relatively low percentage of G1-phase cells because of a shortened duration of G1 [20–22]. This finding has been well documented in several ways [23–25], including by the use of the fluorescent ubiquitination–based cell-cycle indicator (FUCCI) system [26]. Pluripotent stem cells initiate differentiation from G1 phase [22,25,27–30]. This finding links heterogeneous gene expression and cell-cycle progression as shown by RNA sequencing (RNA-seq) of FUCCI-labeled G1 cells [31]. Thus, G1 phase–constrained gene expression may be a possible mechanism for “lineage priming,” particularly in light of G1-associated up-regulation of the epigenetic mark, 5-hydroxymethylcytosine (5-hmC), thought to have a role in gene activation [31–33] and cyclin D–dependent transcription [34].

How single-cell variation influences population behavior and provides the many developmental options available to human embryonic stem cells (hESCs) has been addressed according to a theoretical framework in which single cells have statistical properties that increase the potential of the population [35]. Here, we show that hESCs have high cell-to-cell variation in absolute G1 length, and with increased single-cell variation, a population bias toward neuroectoderm (NE) emerged well before the onset of differentiation. Thus, G1 length distribution patterns of a stem cell population represent a probability density curve that can predict differentiation outcome as a predominantly NE or mesendoderm (ME) population. An hESC population with a short and narrow distribution of G1 lengths was biased toward predominantly ME, whereas a long and wide distribution of G1 lengths biased the pluripotent stem cells toward both NE and ME lineages. WNT is centrally positioned in decisions regarding pluripotency because it can control both self-renewal and differentiation, a property that is likely related to its control over G1 length [36–39]. The control that WNT exerts over G1 length results in a stable, yet dynamic, population. Lineage priming occurs by reducing WNT levels, which promotes differentiation and skews the single-cell distribution of stem cell G1 lengths toward longer time intervals and higher single-cell variation. Furthermore, the effect of G1 lengthening on downstream gene expression operates through increased 5-hmC modification. We propose that the high variation in gene expression across single stem cells represents a large parameter space that can be collapsed to a single-cell G1 length distribution curve, which predicts the population differentiation potential to NE or ME.

## Results

### Cell-to-cell variation in G1 and S/G2/M lengths in an hESC population

To analyze absolute lengths of each phase of the cell cycle at a single-cell level, we used the FUCCI reporter [26]. Based on cell cycle–dependent degradation of chromatin licensing and DNA replication factor 1 (CDT1) and geminin DNA replication inhibitor (GEMININ) proteins, cells show no color in early G1, red in mid/late G1, and green in S/G2/M (S1A Fig). FUCCI hESCs showed high expression of pluripotency genes and activated markers for NE and ME upon differentiation (S1B and S1C Fig). Time-lapse images were taken from the FUCCI hESCs grown in mTeSR1 medium for 24–48 h with 10-min intervals (S1 and S2 Movies). Compared with previous reports [25,31], one obvious difference we found in our data is that early G1 phase is very short—less than one frame (10 min) in most cells (S1D Fig and S3 Movie). This discrepancy is likely due to the use of different fluorescent proteins. Pauklin and colleagues [25] used monomeric Kusabira orange 2 (mKO2) fluorescent protein fused to CDT1 (for G1 phase), whereas we used mcherry. The maturation half-time of mKO2 is much longer (1.2 h) than mcherry’s (15 min) [40,41], which could explain a higher percentage of early G1 cells (no color) in their results. To confirm these results, we derived three independent clonal lines from FUCCI hESCs and observed that almost all cells have a no-color phase shorter than 10 min (S1E Fig). These findings suggest that defining early G1 phase by the absence of fluorescent signal could be influenced by differences in maturation time of fluorescence proteins. The short maturation of mcherry–CDT1 fusion proteins more accurately represents the duration of the entire G1 phase and demonstrates that early G1 contributes very little to overall G1 length.

**Fig 1:**
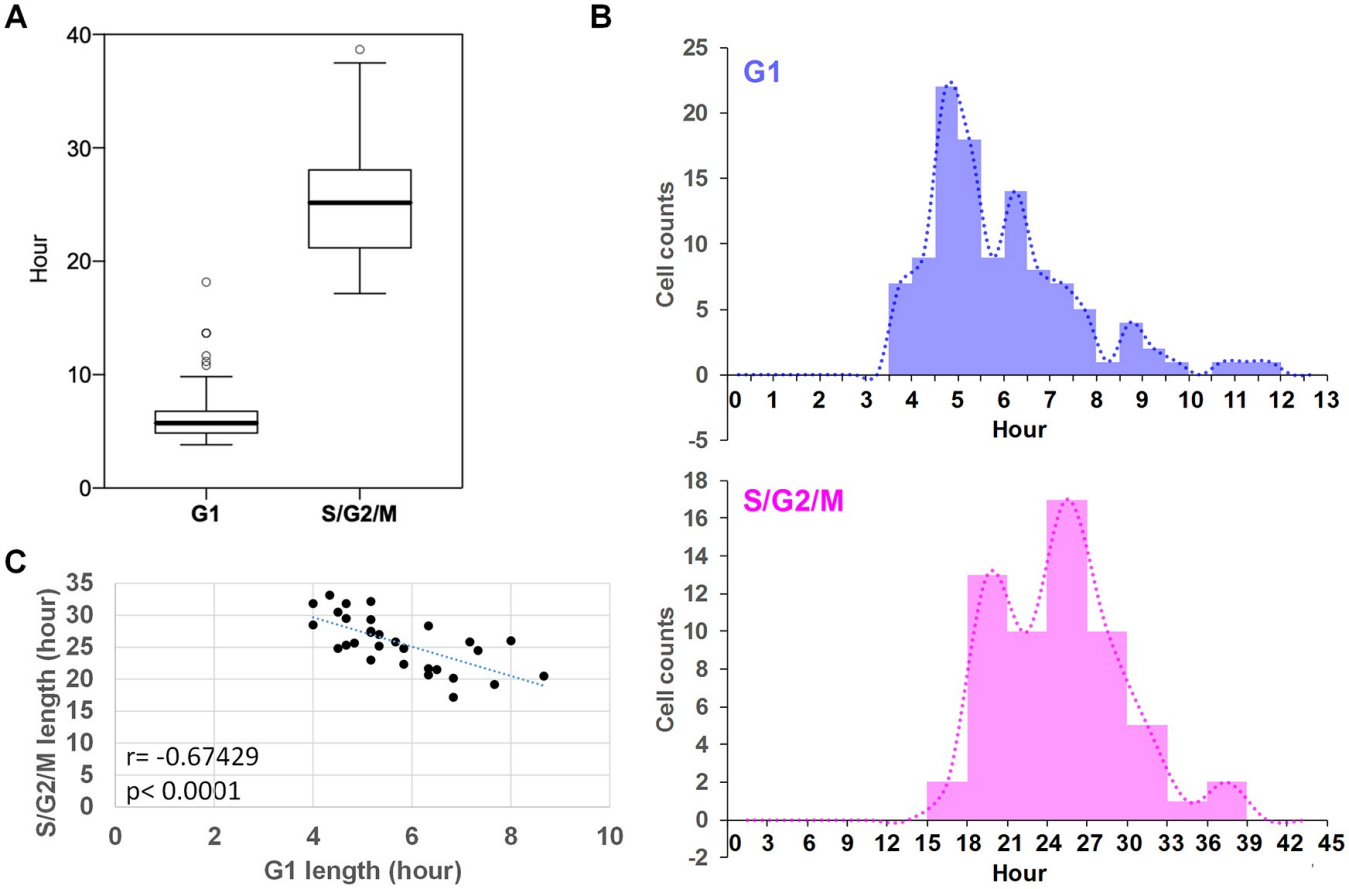
Single-cell heterogeneity in G1 and S/G2/M lengths in self-renewing hESCs. (A) Absolute cell-cycle length of each cell-cycle state in H9 hESCs grown in mTeSR1 medium (*n* = 114 for G1 and *n* = 60 for S/G2/M). (B) Histograms for G1 and S/G2/M lengths. (C) Inverse relationship between G1 and S/G2/M lengths in individual cells (*n* = 30). Both G1 and S/G2/M lengths were measured from the same cell. Data in (C) are a subset of those in (A). Data were collected and pooled from three independent experiments. Underlying data can be found in S1 Data. hESC, human embryonic stem cell.

Individual hESCs showed high variation in the lengths of G1 and S/G2/M phases, with coefficients of variation (CVs) 36.6% and 18.6%, respectively (Fig 1A). G1 lengths ranged from 4 h to 10 h, and S/G2/M lengths ranged from 20 h to 40 h (Fig 1B). Interestingly, G1 lengths inversely correlated with S/G2/M length (*p* < 0.0001) in the same cell (Fig 1C), suggesting that cells with short G1 tend to have long S/G2/M and vice versa. This inverse correlation suggests that individual cells conserve total cell-cycle length through compensatory adjustments of cell-cycle phases. Recently, a compensatory relationship between S- and G2-phase lengths was observed in chordate ascidians during gastrulation [42]. Overall, these results suggest that hESCs have high single-cell heterogeneity in G1 and S/G2/M lengths under self-renewing conditions.

### Distribution patterns of single-cell G1 length determine differentiation outcomes of stem cell populations

**Fig 2:**
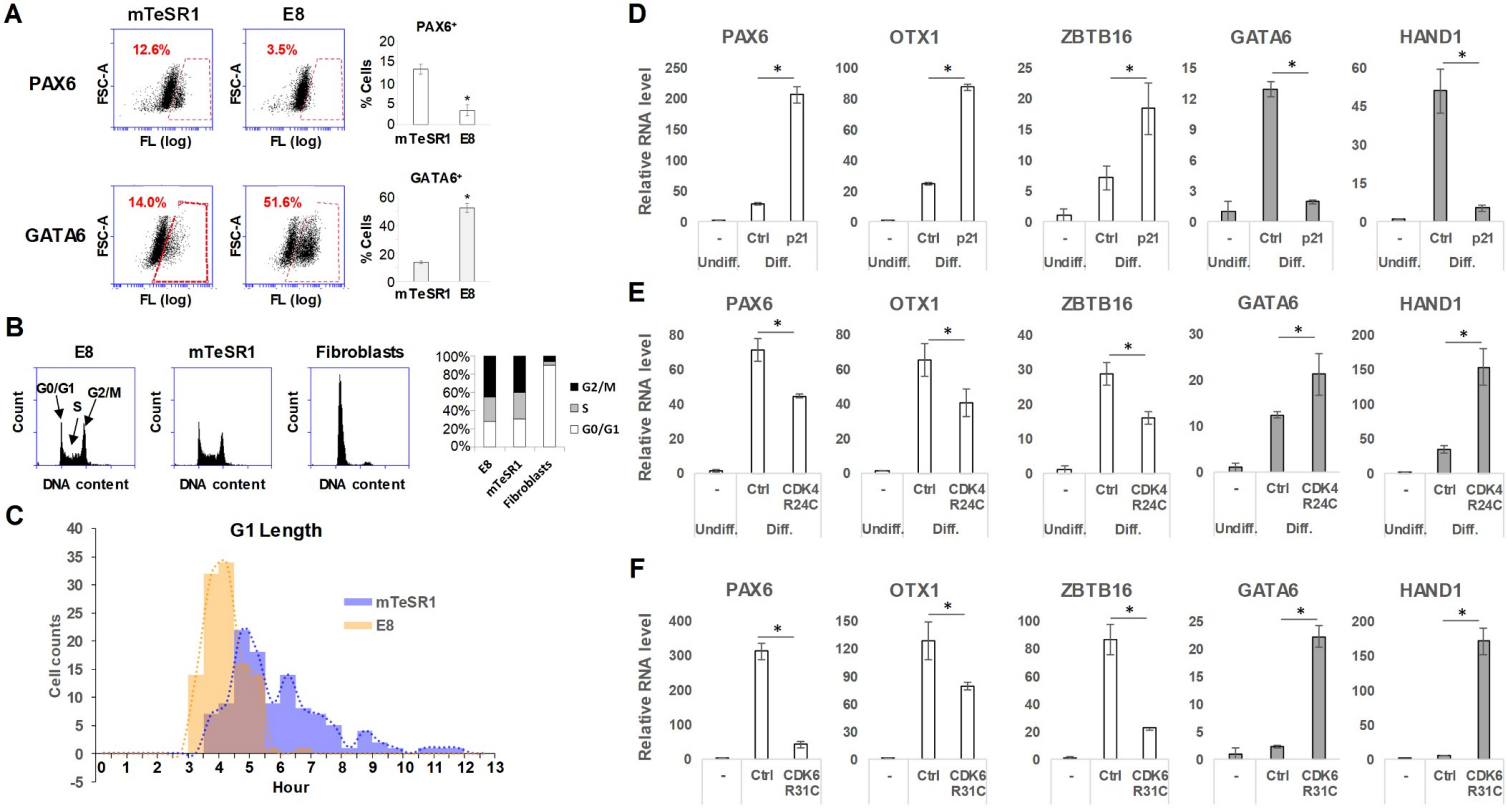
Single-cell distribution patterns of absolute G1 length determine differentiation propensity of hESC populations. (A) Immunoflow cytometry of PAX6 and GATA6 in H9 cells differentiated for 8 d by FGF2 deprivation (*n* = 4). (B) Propidium iodide staining analysis of H9 cells either in E8 or in mTeSR1 media and human dermal fibroblasts (*n* = 4 for E8 and mTeSR1, *n* = 3 for fibroblasts). (C) Histograms for G1 length of H9 cells grown either in E8 or in mTeSR1 (*n* = 112 for E8 and *n* = 114 for mTeSR1 pooled from three independent experiments); U test: *p*-value < 2.2 × 10^−16^, KS test: *p*-value < 2.2 × 10^−16^. (D-F) qPCR analysis of lineage markers in differentiated day 8 H9 cells overexpressing p21 (D), CDK4^R24C^ (E), or CDK6^R31C^ (F) (*n* = 4). Ctrl represents cells transduced with empty lentiviral vectors and treated with the same dose of doxycycline (1 μg/ml). Transgene expression was turned off at the onset of differentiation by doxycycline withdrawal. Error bars represent SD. *p < 0.01 (Student *t* test). Underlying data can be found in S1 Data. CDK, Cyclin-dependent kinase; Ctrl, control; Diff. differentiated; E8, Essential 8; FSC, forward scatter; FGF, Fibroblast growth factor; FL, fluorescence intensity; GATA6, GATA binding protein 6; HAND1, Heart and neural crest derivatives expressed 1; hESC, human embryonic stem cell; KS, Kolmogorov-Smirnov; OTX1, Orthodenticle homeobox 1; PAX6, Paired box 6; qPCR, quantitative PCR; Undiff., undifferentiated; ZBTB16, Zinc finger and BTB domain containing 16.

G1 phase has the potential to make cell fate decisions in response to extracellular cues, whereas cells in S/G2/M phase do not respond to differentiation cues [25,30,31,43]. The different response of each cell-cycle state to differentiation cues is mechanistically supported by evidence that epigenetic modifications are regulated in a cell cycle–dependent manner [31,44–46]. Recent papers nicely showed that gene activation–related epigenetic markers such as 5-hydroxymethylcytosine and histone 3 lysine 4 trimethylation (H3K4me3) accumulated in lineage-specific genes as the G1 phase progresses [31,45,46]. Therefore, we hypothesized that individual hESCs differ in their differentiation potential based upon their absolute G1 lengths. To broach this question, we compared H9 hESCs in two well-defined feeder-free stem cell media, Essential 8 (E8) and mTeSR1. These two different media conditions gave us a unique opportunity to study the correlation between G1 length and differentiation potential in an identical cell line. Consistent with the robust ability of both media to maintain pluripotency, H9 cells showed high expression levels of key pluripotency genes (Octamer-binding transcription factor 4 [OCT4], NANOG, Sex determining region Y-box 2 [SOX2]) under both conditions (S2A Fig). To measure the differentiation potential, we spontaneously differentiated H9 cells in hESC medium (see Materials and methods) without FGF2. In this differentiation condition, H9 cells in E8 exhibited highly biased differentiation toward ME, whereas H9 cells in mTeSR1 differentiated more equally to both NE and ME lineages (Fig 2A). The mRNA levels of lineage markers showed consistent patterns with the flow cytometry data (S2B Fig). Lineage-specific differentiation protocols, dual SMAD inhibition for NE differentiation, and FGF2 and BMP4 for ME differentiation also confirmed that hESCs differ in their differentiation propensity depending on the conditions under which they were grown before differentiation (S2C Fig) [47,48]. Both media conditions (mTeSR1 and E8) showed, as expected, similar cell-cycle patterns, with a low percentage of cells in G1 and a high percentage of cells in S/G2/M (Fig 2B, S2D Fig). These results were further confirmed by using 5-bromo-2′-deoxyuridine (BrdU)/7-amino-actinomycin D (7-AAD), which clearly separates S phase from the others (S2E Fig). When absolute G1 length was measured from single cells, however, a dramatic difference emerged, suggesting that mTeSR1 and E8 represent two distinct distributions (*p* < 2.2 × 10^−16^ by the Kolmogorov-Smirnov test and *p* < 2.2 × 10^−16^ by the Mann-Whitney U test). Cells grown in E8 showed a loss of the long G1 population and consequently a decreased mean G1 length (Fig 2C). Biological replicates displayed similar mean and variation (S2F Fig).

Because the G1/S checkpoint plays a key role in determining G1 length, we measured the expression of cyclins. Consistent with a short average G1 length, H9 cells in E8 medium showed higher expression of cyclins D and E, which facilitate the G1/S transition (S2G and S2H Fig), but not cyclins A and B (S2G and S2H Fig). Given the ME-biased potential of E8 in the context of our spontaneous differentiation condition, we assumed that the population of cells with long G1 might be responsible for the NE lineage preference. To further validate this idea, we compared three well-defined human induced pluripotent stem cell (hiPSC) lines (S3A Fig) [49]. As high variability in differentiation potential was reported in iPSC lines [50–52], the three hiPSC lines have a dramatic difference in differentiation propensity (S3B Fig). Consistent with the hESC data (Fig 2C), highly neurogenic hiPSC1 contained a substantial population of cells with longer G1 compared with hiPSC2 and hiPSC3 (S3C Fig). However, all hiPSC lines maintained similar relative proportions of cells in each cell-cycle state (S3D Fig), emphasizing that relative cell-cycle length is not related to the differentiation potential of human pluripotent stem cells (hPSCs). Rather, variation in length among the same number of cells in G1 establishes differentiation potential.

To test the functional relationship between absolute G1 length and cell fates, we modulated G1 length in self-renewing H9 cells by overexpressing p21 or constitutively active Cyclin-dependent kinases (CDK4^R24C^ and CDK6^R31C^) (S4A Fig). We used a doxycycline-dependent lentiviral system to control the transgene expression. Overexpression of p21 increased the average length of G1, whereas CDK4^R24C^ and CDK6^R31C^ reduced it (S4B Fig). The modulation of absolute G1 length did not affect pluripotency gene expression and the relative cell-cycle patterns (S4A, S4C and S4D Fig), which suggests that hESCs can tolerate high variation in G1 length. Transgene expression was turned off at the onset of differentiation to exclude any potential effect of the transgene on differentiation (S4E Fig). Transient increase of G1 length by p21 further promoted NE derivation at the expense of ME differentiation (Fig 2D). These results were confirmed by abemaciclib, a potent and selective chemical inhibitor of CDK4 and CDK6. Abemaciclib treatment for 18 h before differentiation phenocopied the effect of p21 overexpression (S4F Fig). Transient decrease of G1 length by CDK4^R24C^ and CDK6^R31C^ showed the opposite effect (Fig 2E and 2F). Furthermore, increased G1 length by p21 overexpression in H9 cells grown in E8 medium was sufficient to improve neurogenic potential to the level of those in mTeSR1 at the expense of ME lineage derivation (S4G Fig), suggesting that the difference in differentiation propensity between the two media conditions can be attributed to G1 length. Collectively, these results suggest that G1 length biases the differentiation potential of self-renewing hESCs, and thus, the G1 length distribution patterns determine population fates upon differentiation.

### Asymmetric sister cell G1 duration contributes to heterogeneity in single-cell G1 length

**Fig 3:**
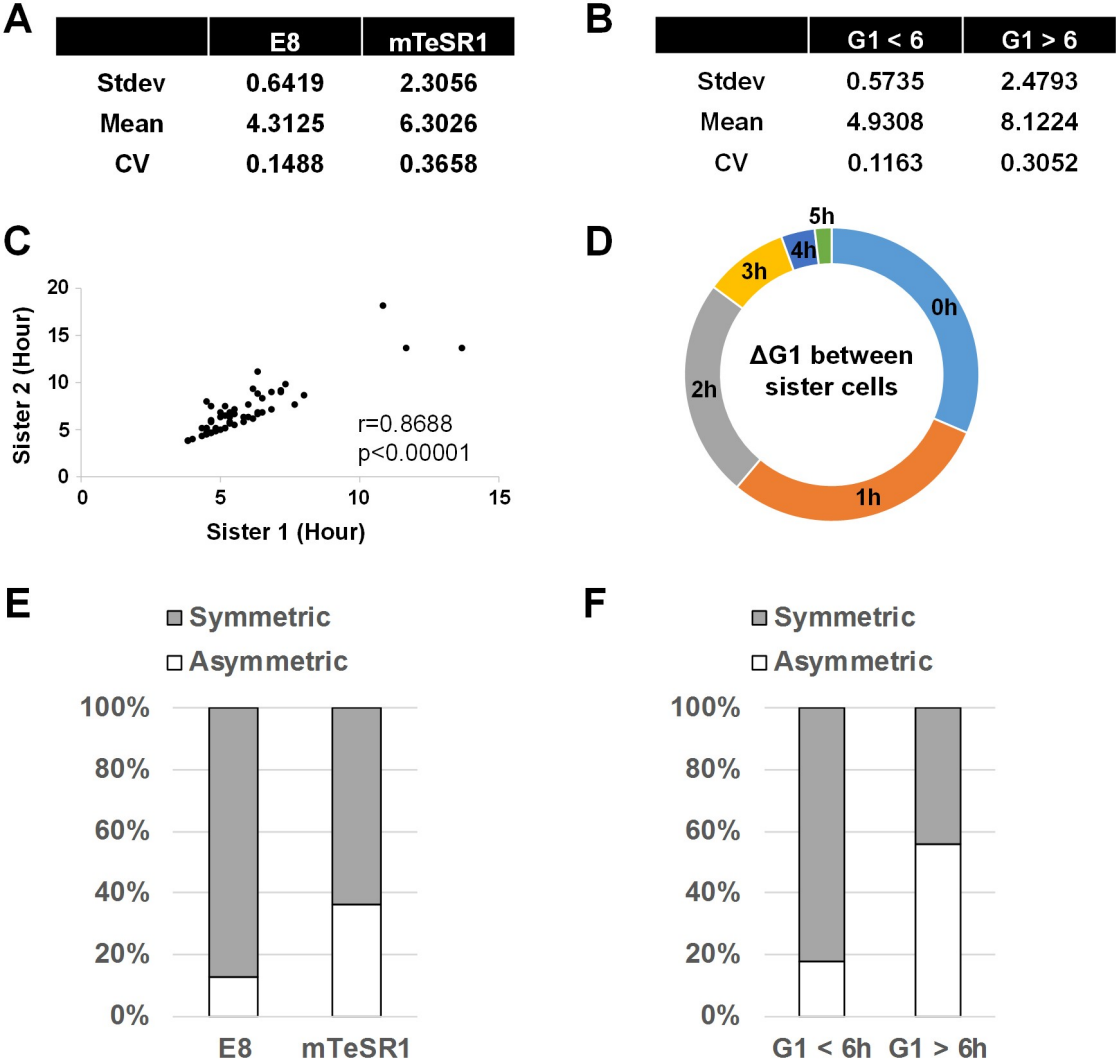
Relationship between G1 length and single-cell variation. (A) Stdev, mean, and CV of the G1 length distributions shown in Fig 2C. (B) Stdev, mean, and CV of the mTeSR1 distribution (Fig 2C) divided by 6-h cutoff of G1 length. (C) Correlation plot of G1 lengths between sister cells (*n* = 55 from three independent experiments). (D) Ring plot showing the difference in G1 lengths between sister cells (*n* = 55 from three independent experiments). (E) Ratio of symmetric and asymmetric sister cell G1 durations in H9 cells grown either in E8 or in mTeSR1 (*n* = 56 for E8 and *n* = 55 for mTeSR1 pooled from three independent experiments). (F) Ratio of symmetric and asymmetric sister cell G1 durations in the mTeSR1 distribution divided by 6-h cutoff of G1 length (*n* = 55 pooled from three independent experiments). Underlying data can be found in S1 Data. CV, coefficient of variation; E8, Essential 8; Stdev, standard deviation.

Cells grown in mTeSR1 have not only an increased mean G1 length but also a greater CV than those of E8 (Fig 3A). The larger variation of G1 length in a highly neurogenic stem cell population was also observed in hiPSC lines (S3C Fig). The CV of hiPSC1 is significantly higher than hiPSC2 and 3 (*p* = 0.0001284737 for hiPSC1 versus hiPSC2 and *p* = 0.01884844 for hiPSC1 versus hiPSC3) [53]. Because the stem cell populations with larger variation also had higher mean G1 length, we analyzed the relationship between G1 length and variation. Single-cell G1 length data of cells grown in mTeSR1 were divided into cells with G1 lengths longer or shorter than 6 h. The 6-h cutoff was chosen because most of the cells in E8 had G1 lengths less than 6 h. Interestingly, cells with G1 < 6 h showed a similar level of G1 length variation compared with those in E8, whereas cells with G1 > 6 h had higher variation (Fig 3B). These data suggest that G1 length is related to single-cell variation.

Next, we sought the source of single-cell variation in G1 length. Interestingly, G1 lengths between sister cells showed a good correlation (Fig 3C), suggesting that sister cells tend to have similar G1 lengths. However, cells that do not share G1 length between sisters are frequent (Fig 3D). Therefore, we hypothesized that single-cell variation in a population might arise from asymmetry of G1 length between sister cells. To test this hypothesis, we measured the difference of G1 length between sister cells (ΔG1) and divided ΔG1 by mean G1 length of sister cells (<G1>). We set ΔG1/<G1> less than 0.2 as symmetric sister cell G1 duration and ΔG1/<G1> greater than 0.2 as asymmetric sister cell G1 duration. Consistent with the large variation of G1 length, H9 cells in mTeSR1 showed a higher percentage of asymmetric sister cell G1 duration than those cells grown in E8 (Fig 3E). This pattern was also observed with sliding cutoffs (S5 Fig), suggesting that the selection of a specific cutoff does not determine the pattern. When the mTeSR1 data were divided into two groups, G1 < 6 h and G1 > 6 h, a higher percentage of asymmetric sister cell G1 duration was observed in cells with G1 > 6 h (Fig 3F). Cells with G1 < 6 h showed a similar percentage of asymmetric sister cell G1 duration compared with those in E8. Overall, these results suggest that long G1 length is related to increased asymmetric sister cell G1 duration and a larger single-cell variation in G1 length.

### WNT/β-catenin pathway controls G1 length distribution patterns

**Fig 4:**
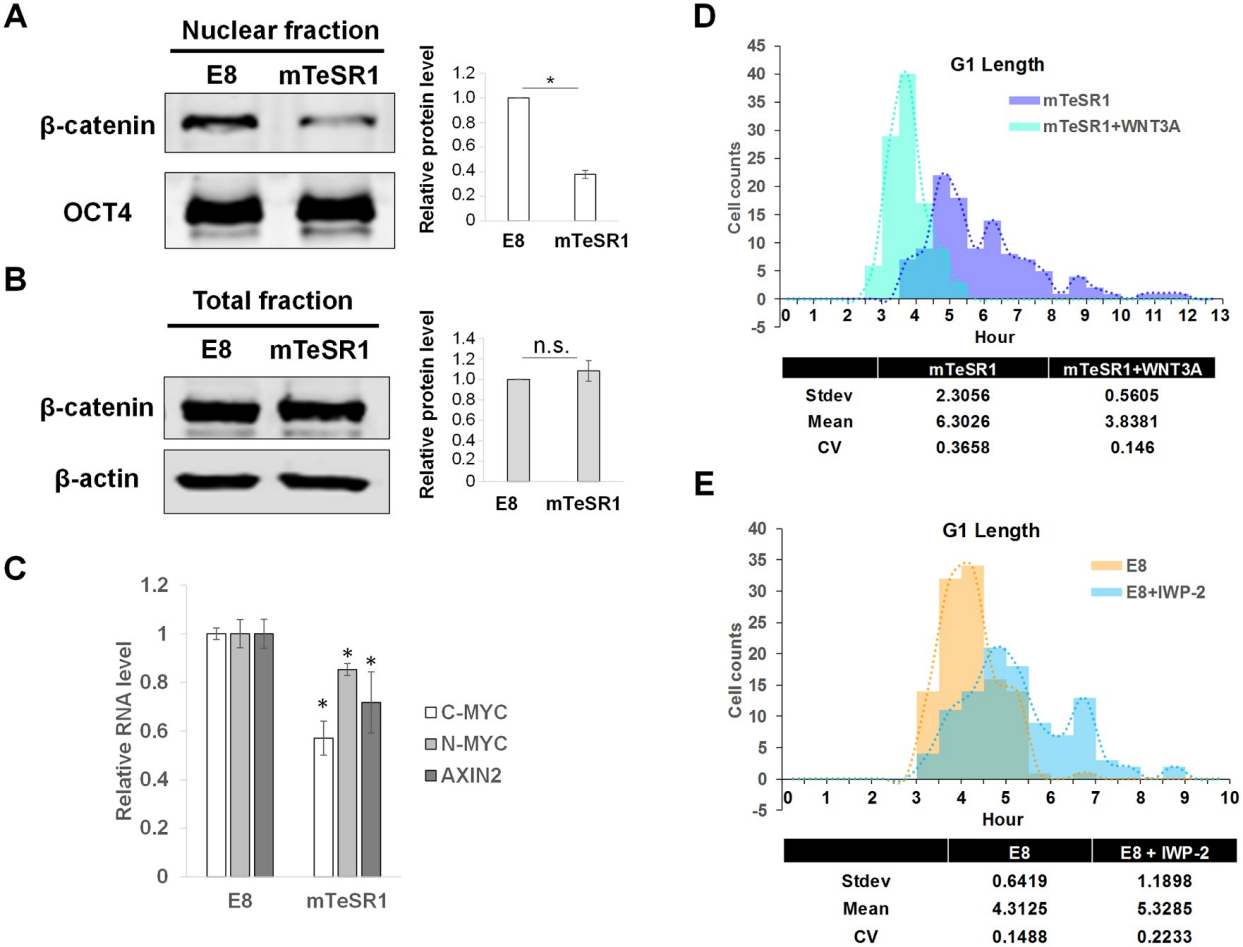
Population WNT levels determine distribution patterns of single-cell G1 length. (A and B) Western blot of β-catenin in nuclear (*n* = 3) and total (*n* = 4) fractions of H9 cells. (C) qPCR analysis of WNT target genes in H9 cells grown either in E8 or in mTeSR1 (*n* = 6). (D) Histograms for G1 length of H9 cells grown in mTeSR1 and treated with recombinant human WNT3A proteins (100 ng/ml) (*n* = 114 for mTeSR1 and *n* = 104 for mTeSR1 + WNT3A pooled from three independent experiments); U test: *p*-value < 2.2 × 10^−16^, KS test: *p*-value = 2.492 × 10^−11^. (E) Histograms for G1 length of H9 cells grown in E8 and treated with IWP-2 (5 μM) (*n* = 112 for E8 and *n* = 104 for E8 + IWP-2 pooled from three independent experiments); U test: *p*-value = 8.282 × 10^−12^, KS test: *p*-value = 1.167 × 10^−9^. Underlying data can be found in S1 Data. AXIN2, Axis inhibition protein 2; C-MYC, c-myc proto-oncogene; CV, coefficient of variation; E8, Essential 8; KS, Kolmogorov-Smirnov; N-MYC, n-myc proto-oncogene; n.s., not significant; OCT4, Octamer-binding transcription factor 4; qPCR, quantitative PCR; Stdev, standard deviation; WNT, Wingless-INT.

To understand the controls over single-cell G1 length variation, we investigated an upstream regulator of G1 length in hESCs, the WNT/β-catenin pathway. This pathway directly controls the expression of many cell-cycle genes and plays a key role in embryonic stem cell (ESC) self-renewal [37–39,54,55]. The down-regulation of WNT/β-catenin drives mouse ESC (mESC) from naïve to primed pluripotency [56]. Furthermore, the unequal distribution of WNT/β-catenin pathway proteins during cell division induces asymmetric division of hESCs [57]. This evidence points to WNT/β-catenin pathway as a strong candidate to control G1 length variation in hESCs. To test this hypothesis, we first analyzed the endogenous activity of WNT/β-catenin pathway in H9 cells grown in either E8 or mTeSR1 by measuring a level of nuclear β-catenin proteins. Higher levels of nuclear β-catenin proteins, but not total proteins, were observed in E8 than in mTeSR1 (Fig 4A and 4B), which was confirmed by the expression of WNT/β-catenin target genes (Fig 4C). A WNT reporter, TOP-flash, also showed a higher mean green fluorescent protein (GFP) intensity in E8 than in mTeSR1 (S6A and S6B Fig). hiPSC2 and hiPSC3 with short G1 also showed higher expression of WNT/β-catenin target genes than hiPSC1 (S6C Fig).

Consistently, WNT activation by recombinant WNT3A proteins was sufficient to shift the G1 length distribution of mTeSR1 to that of E8 without affecting the expression of pluripotency genes and relative cell-cycle patterns (Fig 4D, S6D and S6E Fig). WNT activation induced direct binding of β-catenin around genomic loci of cyclins D1 and E2, which likely increased the expression of those genes and thereby contributed to G1 length shortening (S6F and S6G Fig) [58]. We also confirmed the WNT effect on G1 length in three clonal FUCCI lines (S6H Fig). Furthermore, WNT3A treatment reduced asymmetric sister cell G1 duration and single-cell variation (Fig 4D, S6I Fig). WNT activation also shifted the G1 distribution pattern of hiPSC1 toward those of hiPSC2 and hiPSC3 (S6J Fig). Furthermore, inhibition of WNT production by IWP-2, a Porcupine O-Acyltransferase (PORCN) inhibitor, increased the proportion of cells with longer G1 (Fig 4E, S6K Fig). Collectively, these results suggest that WNT/β-catenin pathway controls the single-cell distribution of G1 length in hESCs.

### Exponential relationship between WNT level and G1 length is captured by a Poisson model

**Fig 5:**
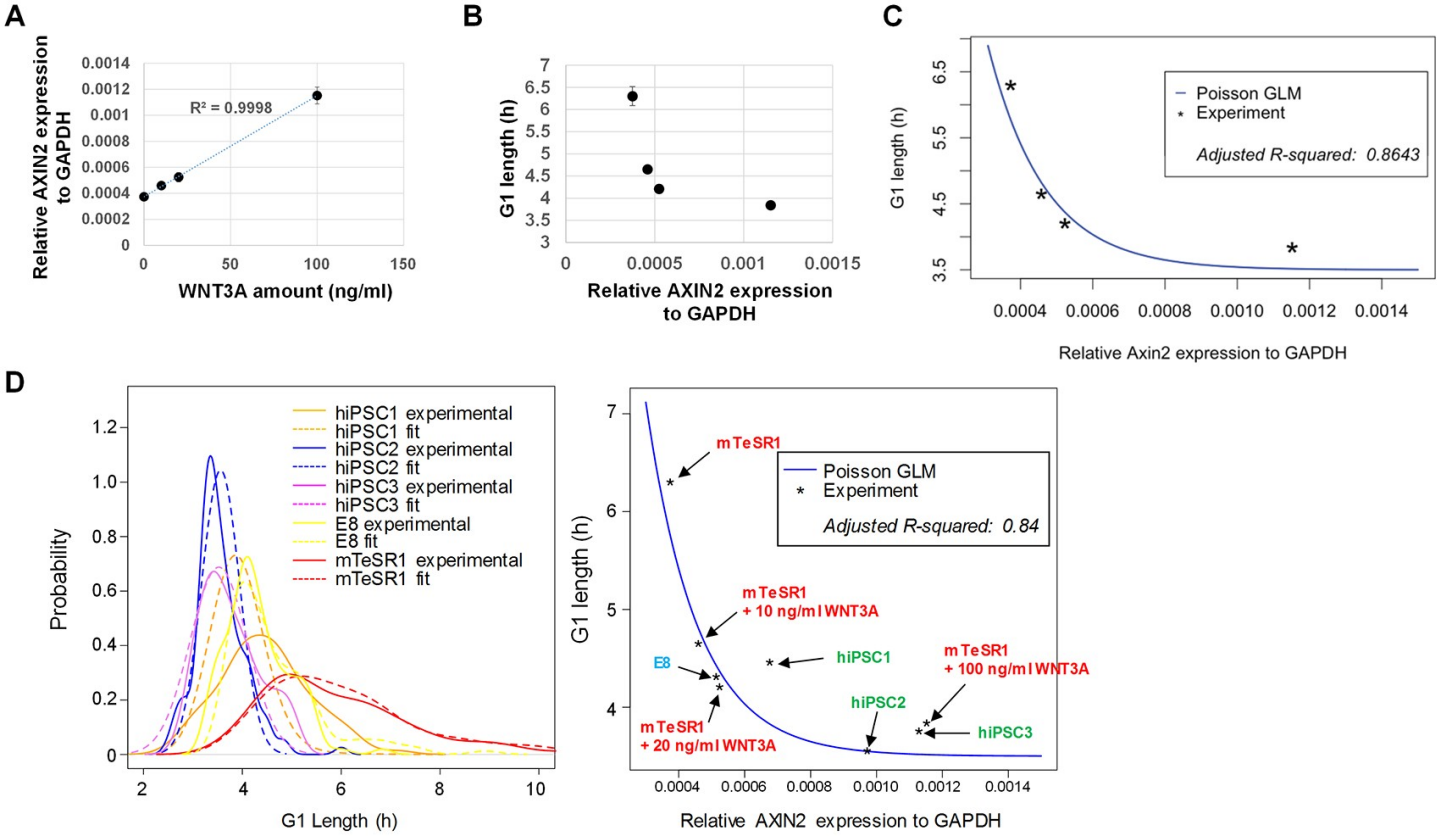
A Poisson model predicts single-cell distribution patterns of G1 length based on population WNT levels. (A) Correlation between AXIN2 expression and the dose of recombinant human WNT3A proteins added to H9 cells in mTeSR1 (*n* = 4). (B) Relationship between average G1 length and population AXIN2 expression in H9 cells treated with various doses of recombinant human WNT3A proteins (*n* = 114 for 0 ng/ml, *n* = 42 for 10 ng/ml, *n* = 48 for 20 ng/ml, and *n* = 104 for 100 ng/ml pooled from two to three independent experiments). Error bars represent SEM. (C) Poisson regression trained with the data in Fig 5B. (D) Poisson regression analysis for combined hESCs and hiPSCs. G1 length is average of multiple single-cell G1 lengths. AXIN2 expression was measured by qPCR (*n* = 4). Underlying data can be found in S1 Data. AXIN2, Axis inhibition protein 2; E8, Essential 8; GAPDH, glyceraldehyde 3-phosphate dehydrogenase; GLM, generalized linear model; hESC, human embryonic stem cell; hiPSC, human induced pluripotent stem cell; qPCR, quantitative PCR; WNT, Wingless-INT.

To gain a better insight of WNT control over G1 length, we analyzed the quantitative relationship between WNT level and G1 length. G1 length was measured with increasing doses of recombinant WNT3A proteins. Axis inhibition protein 2 (AXIN2) expression was used as a marker for endogenous WNT activity because AXIN2 expression is a general indicator of WNT/β-catenin pathway activity [59]. AXIN2 expression showed a linear correlation with the amount of WNT3A proteins added (Fig 5A); hence, WNT3A proteins were not present in saturation conditions. In striking contrast, we observed an exponential relationship between AXIN2 expression and G1 length (Fig 5B). Based on this observation, we pursued a regression model to better understand the nature of G1 length distribution in hESCs (for model details, see Materials and methods). G1 lengths can be modeled as a sequence of exponentially distributed intracellular events. It was reported that a gamma distribution, or a shifted gamma distribution, provides good fits for these types of distributions [60–62]. In particular, plotting G1 length by shifting its value to the origin, such that G1* = G1 − min(G1), we can, to high accuracy, describe G1* with a generalized Poisson distribution (which is a special case of a gamma distribution). We start our analysis by illustrating that G1* ~ Poisson(μ), where μ = <G1*> is the average of G1*. Then, by regressing the experimental data using a Poisson regression as follows:

$$\mu_{i}=g^{-1}(\eta_{i})$$

$$\eta_{i}=\beta_{0}+\beta_{1}x_{i}+\varepsilon_{i},\;{\varepsilon_{i}}_{\;\sim }^{iid}\;N(0,\sigma^{2})$$

where ***g***(.) = log is called “link function,” covariate x is defined as AXIN2 expression for G1* as a response variable (i.e., μ). We used a q-q plot to examine the validity of our regression model (S7A Fig). The above Poisson regression model trained by the hESC data with various doses of WNT3A proteins (Fig 5B) results in approximate model parameters ${\hat{\beta }}_{0}=3.2\;and\;{\hat{\beta }}_{1}=-6,380$ (adjusted R-squared = 0.86) (Fig 5C). Next, this model was tested on all data, which shows a reasonable agreement with the experimental data (adjusted R-squared = 0.84) (Fig 5D). Our analysis showed that each G1 length distribution closely followed a Poisson distribution (Fig 5D, S7A and S7B Fig). Hence, we propose a Poisson distribution for G1* as well as an exponential relation between <G1*> and WNT level as follows:

$$P({G1}^{*})=\frac{\mu^{{G1}^{*}}e^{-\mu }}{{\Gamma (G1}^{*}+1)},$$

where $\mu ={<{G1}^{*}>=e}^{3.2-6,380*Axin2}$, Г: gamma function ∴ $<{G1}^{*}>=e^{-WNT}$

The equation recapitulates the key features of our experimental data in which low WNT levels result in increased mean G1 length and higher CV ($\sqrt{<G1>-\text{min}\left(G1\right)}/<G1>$, see Materials and methods) and, thus, link long G1 length to high single-cell variation. Taken together, these data suggest that single-cell distribution patterns of G1 length can be predicted by our Poisson model based on given population WNT levels, highlighting the important role of WNT in establishing G1 length distribution patterns of stem cell populations.

### G1 length–driven 5-hmC accumulation underlies NE differentiation potential of hESCs

Finally, we sought the molecular mechanism for G1 length control over differentiation outcomes of hESC populations. SMADs are important regulators of hESC differentiation. Recently, it was reported that SMAD2/3 activity changes during the cell cycle [25]. Therefore, we analyzed SMAD2/3 activity by measuring nuclear levels of SMAD2/3 proteins. Despite different G1 lengths, H9 cells showed no significant difference in SMAD2/3 activity under mTeSR1 or E8 media conditions (S8A Fig). Furthermore, G1 shortening by CDK4^R24C^ or CDK6^R31C^ did not affect nuclear shuttling of SMAD2/3 proteins (S8B Fig), suggesting that absolute G1 length is not related to SMAD2/3 activity.

**Fig 6:**
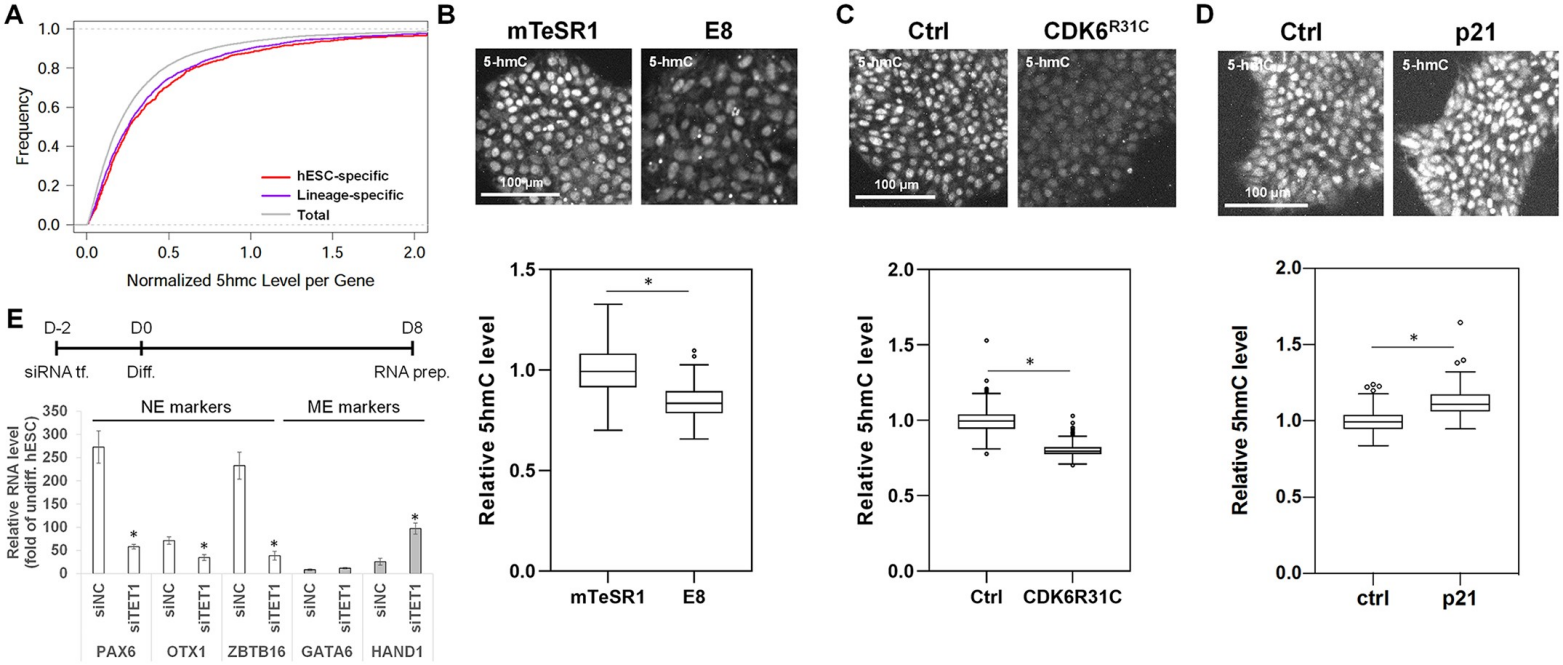
G1 length–driven 5-hmC accumulation is necessary for NE differentiation. (A) Empirical cumulative distribution of 5-hmC levels measured across whole gene bodies (including 1 kb upstream and downstream of genes) in H9 hESCs grown in mTeSR1 (U test: *p*-value < 2.2 × 10^−16^ for Lineage-specific versus Total, *p*-value < 2.2 × 10^−16^ for hESC-specific versus Total, and *p*-value = 0.04956 for Lineage-specific versus hESC-specific). (B) Immunofluorescence assay for 5-hmC in H9 cells grown either in mTeSR1 or in E8 (*n* = 187 for mTeSR1 and *n* = 202 for E8 from three independent experiments). (C and D) Immunofluorescence assay for 5-hmC in H9 cells grown in mTeSR1 overexpressing either CDK6^R31C^ (C) or p21 (D) (panel C: *n* = 222 for Ctrl and *n* = 207 for CDK6^R31C^ pooled from three independent experiments, panel D: *n* = 187 for Ctrl and *n* = 194 for p21 pooled from three independent experiments). (E) qPCR analysis of lineage markers in H9 cells transfected with TET1 siRNA and then differentiated for 8 d (*n* = 4). Error bars represent SD. *p < 0.01 (Student *t* test). Underlying data can be found in S1 Data. 5-hmC, 5-hydroxymethylcytosine; CDK, Cyclin-dependent kinase; Ctrl, control; Diff., differentiated; E8, Essential 8; GATA6, GATA binding protein 6; HAND1, Heart and neural crest derivatives expressed 1; hESC, human embryonic stem cell; ME, mesendoderm; NC, negative control; NE, neuroectoderm; OTX1, Orthodenticle homeobox 1; PAX6, Paired box 6; qPCR, quantitative PCR; RNA prep., RNA preparation; siRNA, small interfering RNA; TET, Ten-eleven translocation; tf., transfection; Total, total protein coding genes; ZBTB16, Zinc finger and BTB domain containing 16.

The 5-hmC converted from DNA methylation (5-methylcystosine [5-mC]) by Ten-eleven translocation (TET) family proteins plays a role in global DNA demethylation [63,64]. The 5-hmC levels in promoters and enhancers correlate with open chromatin structures and gene activation [65–67]. Recently, it was reported that global 5-hmC levels increase during G1 phase in hESCs and that genomic accumulation of 5-hmC is a time-dependent process [31,68]. Based on this evidence, we asked whether 5-hmC could be a key mediator linking G1 length distributions to differentiation outcomes of hESC populations by priming lineage genes. First, we obtained genome-wide 5-hmC profiles in hESCs grown in mTeSR1 at a base resolution level using a unique DNA modification–dependent restriction endonuclease AbaSI (S9A Fig) [68]. The 5-hmC levels were measured across whole gene bodies (including 1 kb upstream and downstream of genes). hESC-specific and lineage-specific genes are defined by gene expression patterns during early hESC differentiation using our published RNA-seq data [69]. Interestingly, when quantitatively comparing 5-hmC levels of lineage-specific genes with total background, we observed significantly higher 5-hmC levels for lineage-specific genes (U test *p*-value < 2.2 × 10^−16^) (Fig 6A). Furthermore, lineage-specific genes showed as high 5-hmC levels as hESC-specific genes even though the expression of lineage-specific genes is much lower than hESC-specific genes in hESCs (U test *p*-value < 2.2 × 10^−16^) (Fig 6A, S9B Fig). These data suggest a potential role of 5-hmC in priming lineage-specific gene activation upon hESC differentiation.

Next, to test whether the length of the G1 phase could influence global levels of 5-hmC in hESCs, we compared global 5-hmC levels of hESCs grown either in E8 or mTeSR1. As expected, mTeSR1 showed a significantly higher level of 5-hmC than E8, consistent with the longer G1 lengths observed in mTeSR1 (Fig 6B). However, there was no significant difference in the expression of TET genes (S9C Fig), which catalyzes conversion of 5-mC to 5-hmC [64]. These results imply that G1 length is tightly linked with 5-hmC levels. To further validate this, we showed that shortening G1 length by expressing constitutively active CDK6^R31C^ decreased 5-hmC levels without affecting 5-mC levels (Fig 6C, S9D Fig). Furthermore, G1-phase lengthening by p21 overexpression increased 5-hmC levels (Fig 6D). Given the neurogenic potential of hESC populations with long single-cell G1 lengths, we tested whether 5-hmC accumulation is essential for NE derivation. To reduce global 5-hmC levels, we used TET1 small interfering RNAs (siRNAs) because TET1 is most abundant in hESCs among TET family genes (S9E–S9G Fig). Decreasing global 5-hmC levels significantly suppressed NE derivation upon differentiation (Fig 6E, S9H and S9I Fig). However, ME differentiation was not affected or even promoted by 5-hmC reduction (Fig 6E, S9H and S9I Fig). Overall, these results suggest G1 length–driven 5-hmC accumulation as a molecular mechanism for NE differentiation potential of hESC populations.

## Discussion

Whereas relative G1 length as reflected in the ratio of S/G2/M phase to G0/G1 phase by propidium iodide fluorescence reflects the population [21,30], absolute G1 length distributions across single cells reflect stem cell duality by establishing a differentiation bias while maintaining self-renewal properties. The maintenance of the stem cell population is buffered against variation in single-cell G1 lengths, whereas, on the other hand, this variation is exploited to generate multiple fates from a stem cell population. Furthermore, we found that single-cell variation in G1 length can be determined by the environmental WNT level—i.e., WNT level in the media—thereby providing an example of how a uniform influence on the entire population can affect single-cell variation. Modeling revealed that population WNT levels account for the G1 length distributions remarkably well, which suggests that in culture, WNT can be an important environmental factor in the establishment of the G1 length distribution. Given the complexity in environmental factors, however, we note that WNT is not likely to be the only factor controlling G1 length and that other signaling molecules potentially cooperate on G1 length regulation. Reduced levels of environmental WNT increase asymmetric sister cell G1 duration and G1 length variation in a stem cell population. Stochasticity of the WNT control over G1 length and “finite number effect” could underlie this observation [70]. G1 length distribution can represent the collective action of multiple genes. Because the effect of WNT levels on G1 length distributions is an exponential function, the steep decline represented by this function will create a sharp boundary of cell fates along a falling gradient of WNT levels.

Given the pleiotropic functions of WNT in development, G1 length control is likely one of many effects induced by WNT. Consistent with extensive cross talk between signaling pathways, we observed that WNT activation increases Transforming growth factor Beta (TGF-β)–SMAD2/3 activity in hESCs (S8C Fig). However, this observation seems to be independent of G1 length because G1 length modulation had no effect on SMAD2/3 activity (S8A and S8B Fig). Therefore, we suggest that WNT activation affects the differentiation propensity of a stem cell population by shifting G1 length distributions, whereas G1 length–independent functions of WNT also contribute to stem cell fate decisions.

Increased G1 length after differentiation has been observed in ESCs and neural stem cells. Furthermore, modulation of G1 length affects stem cell differentiation [21,23,71]. We found that absolute G1 lengths among hESCs bias the differentiation potential of the population. Transient modulation of absolute G1 lengths before differentiation was sufficient to influence differentiation outcome of hESCs. Our model differs from, but does not exclude, the one by Pauklin and colleagues [25]. Pauklin and colleagues [25] used a FUCCI reporter to sort hESCs into early and late G1 cells and found that late G1 cells predominantly differentiate into NE, whereas early G1 cells become ME. By taking a “snapshot” of captured cells, this model does not address the dynamic nature of the population from one generation to the next. Our model explains these experimental results well, given that sorted late G1 populations would be enriched for long G1 cells. Furthermore, the boundary between early and late G1 phases as described by Pauklin and colleagues [25] depends upon the maturation time of fluorescent proteins and, therefore, upon the specific fluorescent proteins used in a reporter system. In contrast, our model adopts an explicit value, absolute length of the G1 phase in single cells. Our single-cell data from time-lapse imaging capture important biological features underlying stem cell differentiation potential, which cannot be seen in a population analysis. For example, Pauklin and colleagues [25] fail to explain dramatic differences in differentiation potential between hESCs grown either in mTeSR1 or in E8 medium with a similar percentage of cells in G1 phase. In contrast, our work identified the important difference in single-cell distributions of absolute G1 length that are responsible for differentiation competency of stem cell populations.

We propose that the absolute lengths of G1 phase establish bias and contribute to the differentiation fates of hPSCs. Therefore, hPSC lines containing predominantly short G1 cells cannot efficiently contribute to NE even though they have a similar number of cells in G1 phase. hPSC lines containing a variable subpopulation of long-G1 cells are capable of deriving NE upon differentiation. These results clearly show that stem cell populations with longer G1 lengths favor NE lineage over ME. On the other hand, the question of differentiation bias in a single cell is more difficult for many reasons; among them is the likely change in G1 length (and consequently bias) from generation to generation. Challenges for imaging over weeks while tracking cell location make this technically difficult. From a cell population perspective, we showed that a G1 length–dependent accumulation of 5-hmC favors NE gene activation and that this epigenetic modification is therefore a likely contributor to population bias. However, G1 length likely affects many cellular processes, and 5-hmC is probably one of many mechanisms potentially linking G1 phase to stem cell fates.

Overall, our findings suggest that, in a cell culture system, WNT-driven G1 length distributions play a key role in determining differentiation potential of stem cell populations. However, the use of this mechanism at the organismal level remains an open question. During development, tissue-specific stem cells change their differentiation potential depending on the context and needs. Dynamic control over G1 length distribution patterns could be an efficient mechanism to regulate differentiation potential of tissue-specific stem cells in dynamic embryonic environments.

## Materials and methods

### Cell culture

H9 cells were maintained in feeder-free conditions on Matrigel (Corning) either in mTeSR1 (Stem Cell Technologies) or E8 (ThermoFisher Scientific) medium. H9 cells were authenticated by short tandem repeat analysis. hiPSC lines (provided by Dr. Yoav Gilad at University of Chicago) were grown on Matrigel in E8 medium [49]. Cells were passaged every 5–6 d by ReLeSR (Stem Cell Technologies). Cells were passaged every 4–5 d by Accutase (ThermoFisher Scientific). HEK293T cells and human dermal fibroblasts were maintained in DMEM supplemented with 10% fetal bovine serum (FBS).

### Differentiation of hESCs and hiPSCs

hESCs and hiPSCs were induced to differentiate on Matrigel in hESC culture medium (DMEM/F12, 15% knockout serum replacement, MEM nonessential amino acid solution, and 0.1 mM β-mercaptoethanol) without FGF2. For NE-directed differentiation, H9 cells were differentiated on Matrigel with hESC culture medium containing Noggin (200 ng/ml) and SB431542 (10 μM) [48]. For ME-directed differentiation, H9 cells were induced to differentiate in mTeSR1 medium containing 10 ng/ml BMP4 [47].

### Generation of FUCCI cell lines and cell cycle–length measurement

H9 hESCs and hiPSCs were transduced with a lentiviral vector expressing the FUCCI reporter (mCherry-hCDT1-T2A-mAG-hGEMININ). Transduced cells were selected by 300 μg/ml neomycin treatment. Cells expressing the FUCCI reporter were imaged by Olympus DSU confocal microscope for 24–48 h with 10-min interval in a chamber with 5% CO_2_ and 37 °C temperature. Duration of each cell-cycle state was measured by manual tracking with the following criteria: G1, from red on to green on; S/G2/M, from red off to green off. To minimize tracking bias, we selected cells randomly regardless of their position in the colony.

### Derivation of clonal FUCCI hESC lines

H9 hESCs were transduced with a lentiviral vector expressing the FUCCI reporter and maintained with 300 μg/ml neomycin. After two rounds of passaging, cells were dissociated with Accutase (ThermoFisher Scientific), FACS sorted using FUCCI-driven GFP signals, and plated onto Matrigel-coated 96-well plates, which were preincubated with CloneR (Stem Cell Technologies) at a density of one cell per well. After 1 wk of media refreshing according to CloneR manufacturer’s protocol, clonal positive wells were selected with the following criteria: (1) only one colony, (2) round/compact colony shape, (3) robust GFP or mCherry expressions across the colony. Each of the clonal FUCCI lines were expanded by at least four rounds of passaging and then used for further experiments.

### Immunofluorescence

Cells were fixed with 4% paraformaldehyde and permeabilized with 0.25% Triton X-100, followed by blocking with 10% FBS in PBS for 1 h. Samples were stained with primary antibodies for OCT4 (Santa Cruz Biotechnology, sc-5279), NANOG (R&D Systems, AF1997), SOX2 (Millipore, AB5603), PAX6 (Santa Cruz Biotechnology, sc-81649), and GATA6 (Cell Signaling Technology, 5851) overnight at 4 °C. Secondary antibody staining was performed for 1 h at room temperature with Alexa Fluor 488-donkey anti-goat IgG, Alexa Fluor 555-donkey anti-mouse IgG, and Alexa Fluor 555-donkey anti-rabbit IgG (ThermoFisher Scientific). For 5-hmC staining, cells were treated with 1.5 M HCl for 30 min at room temperature after 4% paraformaldehyde fixation (antibody: Active Motif, 39769). For 5-mC detection, cells were fixed with ice-cold 70% ethanol for 5 min, followed by 1.5 M HCl for 30 min at room temperature. Samples were blocked with 5% FBS and 0.3% Triton X-100 in PBS for 1 h and then stained with anti-5-mC antibody (Cell Signaling Technology, 28692) diluted in PBS with 1% BSA and 0.3% Triton X-100 overnight at 4 °C. Secondary antibody staining was performed for 1 h at room temperature with Alexa Fluor 555-donkey anti-rabbit IgG (ThermoFisher Scientific). All images were taken using Olympus IX71 fluorescence microscope.

### Western blotting

RIPA lysis buffer (Millipore) was used to lyse cells in the presence of protease inhibitor cocktail (Roche). Protein concentration was measured using Pierce BCA Protein Assay Kit (ThermoFisher Scientific). Same amounts of protein were resolved by 10% SDS-PAGE, followed by transfer to nitrocellulose membranes (GE Healthcare Life Sciences). PBST (0.1% Tween 20 in PBS) containing 5% skim milk was used to block the membranes. Immunoblotting was performed overnight at 4 °C with antibodies for OCT4 (Santa Cruz Biotechnology, sc-9081), cyclin A2 (Cell Signaling Technology, #4656), cyclin B2 (Santa Cruz Biotechnology, sc-28303), cyclin D1 (Millipore, 04–221), cyclin E2 (Santa Cruz Biotechnology, sc-28351), CDK4 (Cell Signaling Technology, 12790), CDK6 (Cell Signaling Technology, 3136), p21 (Cell Signaling Technology, 2947), β-catenin (Millipore, 04–958), Smad2/3 (R&D systems, AF3797), and β-actin (Sigma-Aldrich, A5441). The membranes were stained with secondary antibodies Alexa Flour 680-goat anti-mouse IgG (ThermoFisher Scientific) and IRDye 800CW-goat anti-rabbit IgG (LI-COR) for 1 h at room temperature. Protein bands were visualized with LI-COR Odyssey Imaging System (LI-COR).

### Immunoflow cytometry

Cells were fixed and permeabilized with Fixation/Permeabilization Solution Kit (BD Biosciences). Samples were stained with primary antibodies for PAX6 and GATA6 for 30 min at room temperature, followed by secondary antibody staining with Alexa Fluor 647-donkey anti-rabbit IgG and Alexa Fluor 647-donkey anti-mouse IgG (ThermoFisher Scientific) for 30 min at room temperature. Samples were analyzed by BD Accuri C6 flow cytometry (BD Biosciences).

### Propidium iodide staining

Cells were dissociated into single cells by Accutase (Stem Cell Technologies). Dissociated cells were fixed with cold EtOH for 1 h on ice, followed by RNase treatment. Samples were stained with propidium iodide and analyzed by BD Accuri C6 flow cytometry (BD Biosciences).

### BrdU/7-AAD staining

BrdU/7-AAD staining was conducted using APC BrdU Flow Kit (BD Pharmingen) according to the manufacturer’s protocol. Briefly, H9 cells were treated with BrdU (10 μM) for 15 min, washed with PBS twice, and dissociated into single cells by Accutase. In total, 10^6^ cells per sample were then fixed, permeabilized, and treated with DNaseI (30 μg per 10^6^ cells) for 1 h. Samples were stained with fluorochrome-conjugated anti-BrdU antibody for 20 min and washed, followed by resuspension in 7-AAD solution. At least 10,000 cells per each sample were analyzed by BD Accuri C6 flow cytometry (BD Biosciences).

### Quantitative real-time PCR (qPCR)

Total RNAs were extracted using TRIzol Reagent (ThermoFisher Scientific), followed by reverse transcription with SuperScript III First-Strand Synthesis System (ThermoFisher Scientific). qPCR was performed with Power SYBR Green PCR Master Mix (Applied Biosystems) using QuantStudio 12K Flex Real-Time PCR System. GAPDH was used as a normalization control.

### siRNA transfection

H9 cells were transfected with TET1 siRNAs (ThermoFisher Scientific HSS129586, HSS129587) (100 nM) with RNAiMAX (ThermoFisher Scientific) according to the manufacturer’s protocol.

### Lentiviral production and concentration

Lentiviral vector plasmids were transfected into HEK293T cells with packaging plasmids psPAX2 (Addgene #12260) and pMD2.G (Addgene #12259). Supernatants were collected 48 h posttransfection and filtered through a 0.45-μm filter. Viral supernatants were concentrated by ultracentrifugation.

### Lentiviral vector cloning

To generate a FUCCI lentiviral vector, mCherry-hCDT1-mAG-hGeminin was PCR amplified from the FUCCI reporter (a gift from A. Miyawaki at Brain Science Institute, RIKEN, Wako, Japan) and cloned into pWPI-hPLK2WT-neo (Addgene #35385), replacing hPLK2WT. P21, CDK4^R24C^, and CDK6^R31C^ were PCR amplified from cDNA library of human dermal fibroblasts, pBABE-hygro CDK4 R24C (Addgene #11254), and pcDNA3.1-mouse cdk6 R31C (Addgene #75171), respectively. PCR products were cloned into a doxycycline inducible lentiviral vector expressing eGFP-puro.

### Whole-genome 5-hmC sequencing (Aba-seq)

Samples were prepared as described in scAba-seq with minor modifications [68]. In total, approximately 50,000 cells were suspended in 10 μL of lysis buffer (100 μg Qiagen Protease, 0.04% Triton X-100, 10× NEB CutSmart buffer) and incubated at 50 °C for 15 h, 75 °C for 20 min, and 80 °C for 5 min. In total, 10 μL of 10× NEB CutSmart buffer, 10 U NEB T4-BGT, and 2.5× NEB UDP-Glucose was added to glucosylate 5-hmC sites in the genome, and the sample was incubated at 37 °C for 16 h. Next, 10 μL of protease mix (100 μg Qiagen Protease, 1× NEB CutSmart) was added and incubated at 50 °C for 5 h, 75 °C for 20 min, and 80 °C for 5 min. In all, 10 μL of digestion mix (10 U AbaSI, 1× NEB CutSmart) was added and incubated at 25 °C for 2 h and 65 °C for 20 min. Next, 1 μL of 0.5 μM ds-adaptor was added, followed by 9 μL of ligation mix (2,000 U T4 DNA Ligase, 1× T4-ligase buffer, 3.33 mM ATP), and each sample was incubated at 16 °C for 16 h. The ds-adaptor sequences are described in scAba-seq [68]. DNA cleanup was then performed with 0.825× Agencourt Ampure XP beads and eluted in 25 μL of nuclease-free water. The samples were vacuum centrifuged to a volume of 8 μL. In all, 12 μL of in vitro transcription mix was added (2 μL of each ribonucleotide, 2 μL of T7 buffer, 2 μL T7 enzyme mix) and incubated at 37 °C for 13 h. Library preparation starting from amplified RNA was performed as described in the CEL-Seq2 protocol with the following minor modifications [72]. RNA was fragmented by adding 5 μL of fragmentation buffer (200 mM Tris-acetate [pH 8.1], 500 mM KOAc, 150 mM MgOAc) at 94 °C for 2 min and immediately placed on ice. In total, 2.5 μL of fragmentation stop buffer (0.5 M EDTA) was added to quench the reaction. Next, RNA cleanup was performed using 0.825× Agencourt RNAClean beads and finally resuspended in 22 μL of nuclease-free water. Thereafter, all library preparation steps were performed as described in the CEL-Seq2 protocol [72]. The 5-hmC data analysis pipeline is described in scAba-seq [68]. The 5-hmC site annotation was performed using HOMER software [73]. The 5-hmC level per transcript was quantified by the total number of 5-hmC sites per bin size (gene body and 1 kb upstream and downstream of each transcript) and then normalized with the total number of CG sites for the same bin size. Normalized 5-hmC levels of different transcript isoforms were summed as the 5-hmC level for an individual gene for the following comparison. Lineage genes were obtained based on their expression and significantly up-regulated in either NE or ME from our previously published paper [69]. Likewise, significantly higher expression of genes in hESCs versus both NE and ME were considered as hESC-specific genes. Genome-wide 5-hmC data are available in Gene Expression Omnibus (GEO): GSE113236.

### Poisson model

We start our analysis by proposing a Poisson regression for G1 length as response variable and Axin2 expression as model covariate. For our G1 length to follow a Poisson distribution, var(G1) = <G1> must hold true, but this was not a valid assumption for either hESC or hiPSC. Therefore, we equally shift all G1 length values toward the origin because a Poisson distribution P(x|μ) should be defined for all x > 0. We define G1* as shifted G1 toward the origin G1* = G1 − min(G1), where min(G1) is a single constant obtained for G1 values of the whole data set {hiPSC, hESC}. As a result, the transformed G1 (i.e., G1*) approximately has the mentioned Poisson property var(G1*) ≈ <G1*>. We then used a generalized linear model with g(.) = log(.) as link function (assuming that WNT levels in single cells are independent of each other and their measurements follow a normal distribution) as follows:

$$\mu_{i}=g^{-1}(\eta_{i})$$

$$\eta_{i}=\beta_{0}+\beta_{1}x_{i}+\varepsilon_{i},\;{\varepsilon_{i}}_{\;\sim }^{iid}\;N(0,\sigma^{2})$$

where ***g***(.) = log is called “link function,” resulting in approximate model variables ${\hat{\beta }}_{0}=3.2,\;{\hat{\beta }}_{1}=-6.38\times 1,000$. The higher-order terms (e.g., β_2_x^2^) cannot explain a significant variation in the observations and are neglected to avoid overfitting (*p* > 0.1).

We compare the quantiles log(G1*) with the quantiles of a normal distribution to assess the assumptions of our model in a q-q plot (S7A Fig). This analysis demonstrates that a normality assumption for ƞ is reasonable. Next, we use a Box-Cox transformation (S7B Fig) to show that the log transformation of G1* results in the closest to normal distribution among all of the power transformations (λ_max_ = 0.124~0 and thus, log transformation is the best candidate among all power transformations).

Given G1* ~ Poisson(μ*) (and thus, μ* = var(G1*) = <G1*>), the CV of G1 can be found as follows:

$$CV=\frac{\sigma }{\mu }=\frac{\sigma *}{\mu }=\frac{\sqrt{\mu *}}{\mu }=\frac{\sqrt{\mu -min(G1)}}{\mu }$$

where σ and σ* are the standard deviations of G1 and G1*. As a result, we conclude that the G1 distribution depends on WNT levels such that

- high WNT levels reduce the average G1 in a cell population and
- a population expressing higher WNT levels corresponds to having more homogenous G1 length distributions.

### Statistical analysis

When two groups were compared, a two-tailed Student *t* test was used to determine statistical significance. *p*-Values of less than 0.05 were considered significant. When comparing two distributions of G1 length, the Kolmogorov-Smirnov test and the Mann-Whitney U test were used.

## Supporting information

S1 Fig H9 hESC line expressing the FUCCI reporter. (A) Representative image of FUCCI H9 cells in each cell-cycle state from multiple independent experiments. (B) qPCR analysis of pluripotency genes in FUCCI-expressing H9 cells (*n* = 4). (C) qPCR analysis of lineage markers in FUCCI H9 cells differentiated for 8 d by FGF2 deprivation (*n* = 4). (D) Representative images of FUCCI H9 cells undergoing cell division. (E) Representative images of clonal FUCCI lines undergoing cell division. The duration of no-color phase was measured by live-cell imaging (*n* = 30 for each clone). Error bars represent SD. *p < 0.01 (Student *t* test). Underlying data can be found in S2 Data. FGF, Fibroblast growth factor; FUCCI, fluorescent ubiquitination–based cell-cycle indicator; hESC, human embryonic stem cell; qPCR, quantitative PCR.
(TIF)

S2 Fig G1 length distribution predicts differentiation outcomes of hESCs. (A) qPCR analysis of pluripotency genes in H9 cells grown either in E8 or in mTeSR1 (*n* = 4). (B) qPCR analysis of lineage markers in H9 cells differentiated by FGF2 deprivation for 7 d (*n* = 4). (C) qPCR analysis of lineage markers in H9 cells differentiated for 5 d to NE (dual SMAD inhibition) or ME (FGF2&BMP4) lineage (*n* = 4). (D) FUCCI reporter in H9 cells grown either in E8 or in mTeSR1. Representative images were shown from three independent experiments. (E) BrdU/7-AAD staining and flow cytometry analysis of H9 cells grown either in E8 or in mTeSR1 media (*n* = 3). (F) G1 length data of biological replicates in Fig 2C. (G and H) qPCR (G) and western blot (H) analyses of cyclins in H9 cells grown either in E8 or in mTeSR1 (*n* = 4 for qPCR and *n* = 3~4 for western blot). Error bars represent SD. *p < 0.01 (Student *t* test). Underlying data can be found in S2 Data. 7-AAD, 7-amino-actinomycin D; BMP, Bone morphogenetic protein; BrdU, 5-bromo-2′-deoxyuridine; E8, Essential 8; FGF, Fibroblast growth factor; FUCCI, fluorescent ubiquitination–based cell-cycle indicator; hESC, human embryonic stem cell; ME, mesendoderm; NE, neuroectoderm; qPCR, quantitative PCR.
(TIF)

S3 Fig G1 length distribution predicts differentiation outcomes of hiPSCs. (A) Immunofluorescence of pluripotency genes in hiPSC lines grown in E8 medium. Representative images were shown from three independent experiments. (B) qPCR analysis of lineage markers in hiPSC lines differentiated for 7 d by FGF2 deprivation (*n* = 4). (C) Histograms for G1 length of hiPSC lines (*n* = 100 for hiPSC1, *n* = 120 for hiPSC2, and *n* = 108 for hiPSC3 pooled from two to three independent experiments); U test: *p*-value = 2.242 × 10^−14^ for hiPSC1 versus hiPSC2, *p*-value = 3.395 × 10^−9^ for hiPSC1 versus hiPSC3; KS test: *p*-value = 3.064 × 10^−14^ for hiPSC1 versus 2, *p*-value = 3.209 × 10^−7^ for hiPSC1 versus 3. (D) Propidium iodide staining analysis of hiPSC lines (*n* = 3). Error bars represent SD. *p < 0.01 (Student *t* test). Underlying data can be found in S2 Data. E8, Essential 8; FGF, Fibroblast growth factor; hiPSC, human induced pluripotent stem cell; KS, Kolmogorov-Smirnov; qPCR, quantitative PCR.
(TIF)

S4 Fig Modulation of G1 length affects differentiation propensity of stem cell populations. (A) Western blot of p21, CDK4, CDK6, and OCT4 in H9 cells expressing p21, CDK4^R24C^, or CDK6^R31C^. Representative images were shown from two independent experiments. (B) Average G1 length of FUCCI H9 cells expressing p21, CDK4^R24C^, or CDK6^R31C^ (*n* = 54 for ctrl, *n* = 43 for p21, *n* = 52 for CDK4^R24C^, and *n* = 54 for CDK6^R31C^ pooled from two independent experiments), ^&^*p* < 0.01 (U test); ^§^*p* < 0.01 (KS test). (C) qPCR analysis of pluripotency genes in H9 cells expressing p21, CDK4^R24C^, or CDK6^R31C^ (*n* = 4). (D) Propidium iodide staining analysis of H9 cells expressing p21, CDK4^R24C^, or CDK6^R31C^ (*n* = 3). (E) Dox-induced transgene expression and shutdown after Dox withdrawal in H9 cells transduced with p21, CDK4^R24C^, or CDK6^R31C^ lentiviral vectors. Relative protein levels were analyzed by western blot assay (*n* = 3). (F) Immunofluorescence assay for PAX6 and GATA6 in H9 cells treated with Abemaciclib (0.5 μM) or vehicle (DMSO) for 18 h and then differentiated for 8–10 d. Representative images were shown from two independent experiments. (G) qPCR analysis of lineage markers in differentiated day 8 H9 cells overexpressing p21 (*n* = 4). Transgene expression was turned off at the onset of differentiation by Dox withdrawal. Error bars represent SD. ^#^*p* < 0.05, *p < 0.01 (Student *t* test). Underlying data can be found in S2 Data. CDK, Cyclin-dependent kinase; ctrl, control; Dox, doxycycline; FUCCI, fluorescent ubiquitination–based cell-cycle indicator; GATA6, GATA binding protein 6; KS, Kolmogorov-Smirnov; OCT4, Octamer-binding transcription factor 4; PAX6, Paired box 6; qPCR, quantitative PCR.
(TIF)

S5 Fig Ratio of asymmetric sister cell G1 duration in H9 cells grown either in E8 or in mTeSR1. Difference in G1 length between sister cells (ΔG1) was divided by mean G1 length (<G1>) between sister cells. Asymmetric sister cell G1 duration was defined by ΔG1/<G1> values with various cutoffs (*n* = 56 for E8 and *n* = 55 for mTeSR1 pooled from three independent experiments). Underlying data can be found in S2 Data. E8, Essential 8.
(TIF)

S6 Fig WNT/β-catenin pathway controls G1 length distribution patterns. (A) Validation of TOP-flash reporter by recombinant WNT3A treatment. Representative images were shown from three independent experiments. (B) TOP-flash activity in H9 cells grown either in E8 or in mTeSR1 (*n* = 3). (C) qPCR analysis of WNT target genes in hiPSC lines grown in E8 (*n* = 4). (D) Propidium iodide staining analysis of H9 cells in mTeSR1 and treated with recombinant human WNT3A proteins (100 ng/ml) (*n* = 3). (E) qPCR analysis of pluripotency genes in H9 cells grown in mTeSR1 and treated with recombinant human WNT3A proteins (100 ng/ml) (*n* = 3~4). (F) Analysis of ChIP-seq peaks on the genomic loci of cyclins D1 and E2 with or without WNT3A treatment (GSE64758). Arrows represent genes. (G) qPCR analysis of cyclins D and E in H9 cells grown in mTeSR1 and treated with recombinant human WNT3A proteins (100 ng/ml) (*n* = 4). (H) G1 length of three clonal FUCCI lines treated with recombinant WNT3A (100 ng/ml) (*n* = 30 for each sample). *U test *p*-value < 0.0001. (I) Ratio of symmetric and asymmetric sister cell G1 durations in H9 cells grown in mTeSR1 and treated with recombinant human WNT3A proteins (100 ng/ml) (cutoff: ΔG1/<G1> = 0.2, *n* = 55 for untreated and *n* = 52 for WNT3A pooled from three independent experiments). (J) Histograms for G1 length of hiPSC1 grown in E8 and treated with recombinant human WNT3A proteins (100 ng/ml) (*n* = 100 for hiPSC1 pooled from three independent experiments and *n* = 86 for hiPSC1 + WNT3A pooled from two independent experiments); U test: *p*-value < 2.2 × 10^−16^, KS test: *p*-value < 2.2 × 10^−16^. (K) qPCR analysis for AXIN2 expression in H9 cells treated with 5 μM IWP-2 (*n* = 4). Error bars represent SD. *p < 0.01 (Student *t* test). Underlying data can be found in S2 Data. ChIP-seq, chromatin immunoprecipitation followed by sequencing; E8, Essential 8; FUCCI, fluorescent ubiquitination–based cell-cycle indicator; hiPSC, human induced pluripotent stem cell; KS, Kolmogorov-Smirnov; MFI, mean fluorescence intensity; qPCR, quantitative PCR; WNT, Wingless-INT.
(TIF)

S7 Fig Validation of the Poisson model. (A) q-q plots comparing the quantiles of log(G1*) with the quantiles of a normal distribution. (B) Box-Cox transformation of G1*. Underlying data can be found in S2 Data.
(TIF)

S8 Fig G1 length modulation does not affect SMAD activity in hESCs. (A) Western blot of SMAD2/3 in nuclear and total fractions of H9 cells grown either in E8 or in mTeSR1 (*n* = 4). (B) Western blot of SMAD2/3 in nuclear and total fractions of H9 cells overexpressing CDK4^R24C^ or CDK6^R31C^ (*n* = 3). (C) Western blot of SMAD2/3 in nuclear and total fractions of H9 cells treated with 100 ng/ml of WNT3A for 24 h (*n* = 3). Error bars represent SD. *p < 0.01 (Student *t* test). Underlying data can be found in S2 Data. CDK, Cyclin-dependent kinase; E8, Essential 8; hESC, human embryonic stem cell; WNT, Wingless-INT.
(TIF)

S9 Fig G1 length–driven global 5-hmC levels affect differentiation propensity of stem cell populations. (A) Pie plot for annotated genome categories of 5-hmC sites detected in H9 hESCs grown in mTeSR1. (B) Gene expression patterns of hESC-specific and lineage-specific genes in H9 hESCs (U test: *p*-value < 2.2 × 10^−16^) (GSE69982). (C) qPCR analysis of TET1, TET2, and TET3 in H9 cells grown either in E8 or mTeSR1 (*n* = 4). (D) Immunofluorescence assay for 5-mC in H9 cells grown in mTeSR1 expressing CDK6^R31C^. Representative images were shown from three independent experiments. (E) Gene expression levels of TET1, TET2, and TET3 in hESCs (GSE69982). (F) qPCR analysis of TET1 in H9 cells transfected with siRNAs (*n* = 3). (G) Immunofluorescence assay for 5-hmC in H9 cells transfected with siRNAs. Representative images were shown from three independent experiments. (H) Immunofluorescence assay for PAX6 and GATA6 in H9 cells transfected with siRNAs and then differentiated for 9 d. Representative images were shown from three independent experiments. (I) qPCR analysis of lineage markers in H9 cells grown in mTeSR1, transfected with TET1 siRNA #2, and then differentiated for 8 d without FGF2 (*n* = 4). Error bars represent SD. *p < 0.01 (Student *t* test). Underlying data can be found in S2 Data. 5-hmC, 5-hydroxymethylcytosine; 5-mC, 5-methylctosine; CDK, Cyclin-dependent kinase; E8, Essential 8; FGF, Fibroblast growth factor; GATA6, GATA binding protein 6; hESC, human embryonic stem cell; PAX6, Paired box 6; qPCR, quantitative PCR; siRNA, small interfering RNA; TET, ten-eleven translocation.
(TIF)

S1 Table qPCR primers used in this study. qPCR, quantitative PCR.
(DOCX)

S1 Data Numerical values used in figures. (XLSX)

S2 Data Numerical values used in Supporting information figures. (XLSX)

S1 Movie Time-lapse imaging of FUCCI hESCs in mTeSR1. FUCCI, fluorescent ubiquitination–based cell-cycle indicator; hESC, human embryonic stem cell.
(AVI)

S2 Movie Tracked FUCCI hESCs in mTeSR1. FUCCI, fluorescent ubiquitination–based cell-cycle indicator; hESC, human embryonic stem cell.
(AVI)

S3 Movie FUCCI hESCs undergoing M to G1 phase transition. FUCCI, fluorescent ubiquitination–based cell-cycle indicator; hESC, human embryonic stem cell.
(AVI)

## Abbreviations

5-hmC: 5-hydroxymethylcytosine
5-mC: 5-methylcytosine
7-AAD: 7-amino-actinomycin D
AXIN2: Axis inhibition protein 2
BMP: Bone morphogenetic protein
BrdU: 5-bromo-2′-deoxyuridine
CDK: Cyclin-dependent kinase
CDT1: chromatin licensing and DNA replication factor 1
C-MYC: c-myc proto-oncogene
CV: coefficient of variation
E8: Essential 8
ESC: embryonic stem cell
FGF: Fibroblast growth factor
FL: fluorescence intensity
FSC: forward scatter
FUCCI: fluorescent ubiquitination–based cell-cycle indicator
GEMININ: geminin DNA replication inhibitor
GFP: green fluorescent protein
H3K4me3: histone 3 lysine 4 trimethylation
HAND1: Heart and neural crest derivatives expressed 1
hESC: human ESC
hiPSC: human iPSC
hPSC: human pluripotent stem cell
iPSC: induced pluripotent stem cell
ME: mesendoderm
mESC: mouse ESC
mKO2: monomeric Kusabira orange 2
n.s.: not significant
NE: neuroectoderm
N-MYC: n-myc proto-oncogene
NODAL: Nodal growth differentiation factor
OCT4: Octamer-binding transcription factor 4
OTX1: Orthodenticle homeobox 1
PORCN: Porcupine O-Acyltransferase
RNA-seq: RNA sequencing
siRNA: small interfering RNA
SOX2: Sex determining region Y-box 2
TET: Ten-eleven translocation
TGF-β: Transforming growth factor beta
WNT: Wingless-INT
ZBTB16: Zinc finger and BTB domain containing 16
