# Supplementary figures and images for: Control over single-cell distribution of G1 lengths by WNT governs pluripotency
Source: PLoS Biol. 2019 Sep 26;17(9):e3000453. doi: 10.1371/journal.pbio.3000453 (PMC6782112; doi:10.1371/journal.pbio.3000453)

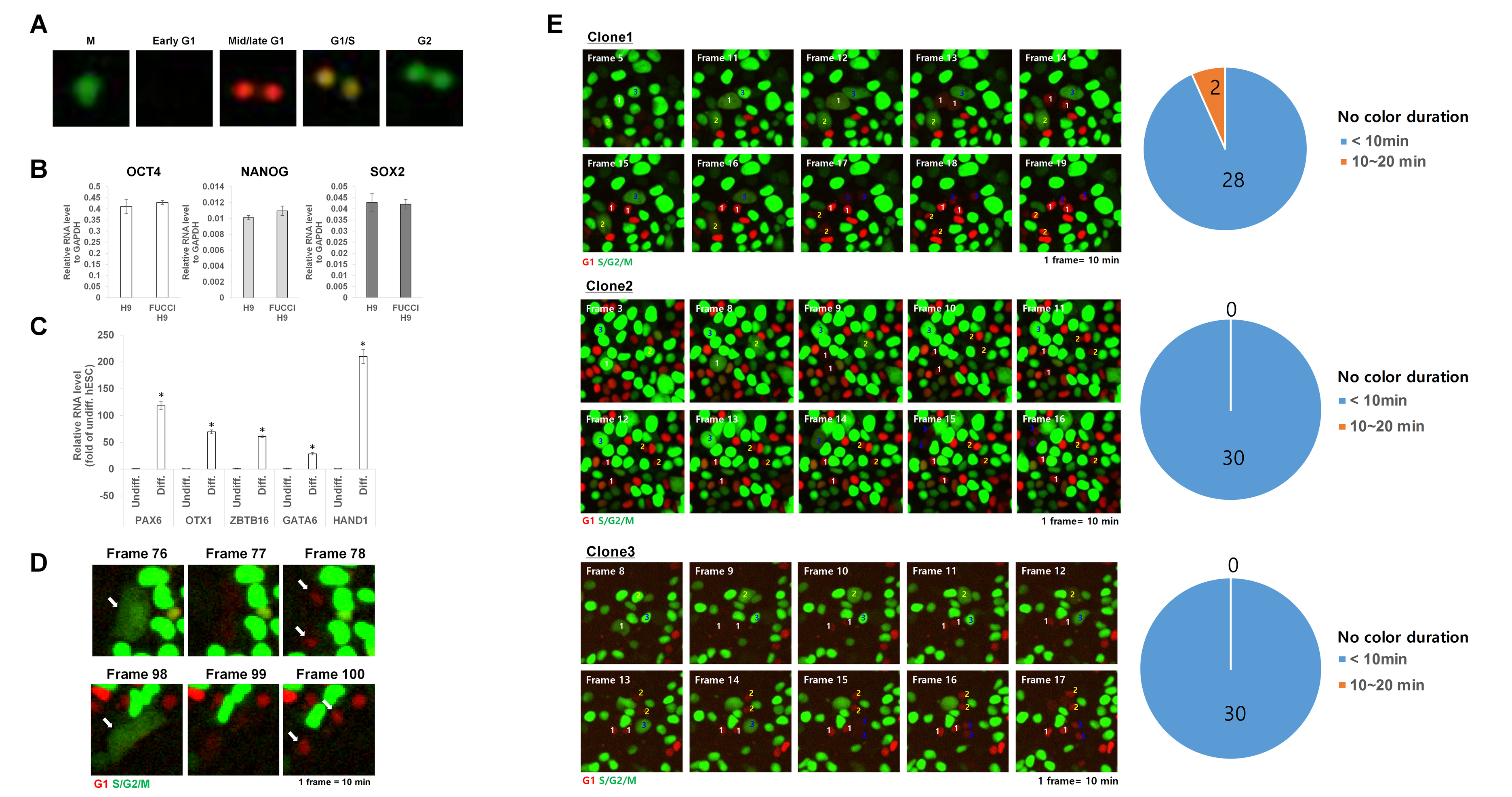

Supplement: S1 Fig — (A) Representative image of FUCCI H9 cells in each cell-cycle state from multiple independent experiments. (B) qPCR analysis of pluripotency genes in FUCCI-expressing H9 cells (n = 4). (C) qPCR analysis of lineage markers in FUCCI H9 cells differentiated for 8 d by FGF2 deprivation (n = 4). (D) Representative images of FUCCI H9 cells undergoing cell division. (E) Representative images of clonal FUCCI lines undergoing cell division. The duration of no-color phase was measured by live-cell imaging (n = 30 for each clone). Error bars represent SD. *p < 0.01 (Student t test). Underlying data can be found in S2 Data. FGF, Fibroblast growth factor; FUCCI, fluorescent ubiquitination–based cell-cycle indicator; hESC, human embryonic stem cell; qPCR, quantitative PCR. (TIF) [file pbio.3000453.s001.tif]

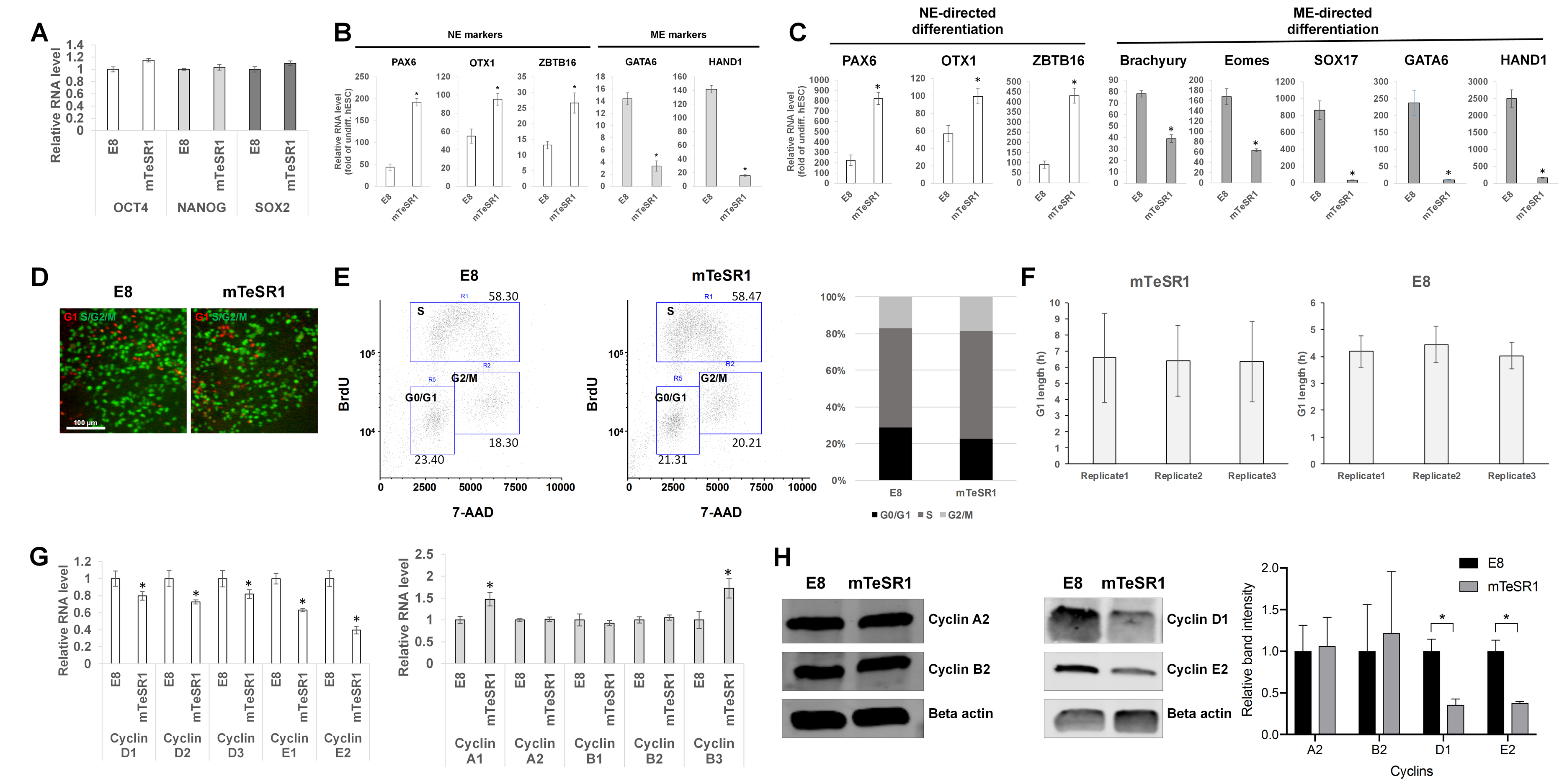

Supplement: S2 Fig — (A) qPCR analysis of pluripotency genes in H9 cells grown either in E8 or in mTeSR1 (n = 4). (B) qPCR analysis of lineage markers in H9 cells differentiated by FGF2 deprivation for 7 d (n = 4). (C) qPCR analysis of lineage markers in H9 cells differentiated for 5 d to NE (dual SMAD inhibition) or ME (FGF2&BMP4) lineage (n = 4). (D) FUCCI reporter in H9 cells grown either in E8 or in mTeSR1. Representative images were shown from three independent experiments. (E) BrdU/7-AAD staining and flow cytometry analysis of H9 cells grown either in E8 or in mTeSR1 media (n = 3). (F) G1 length data of biological replicates in Fig 2C. (G and H) qPCR (G) and western blot (H) analyses of cyclins in H9 cells grown either in E8 or in mTeSR1 (n = 4 for qPCR and n = 3~4 for western blot). Error bars represent SD. *p < 0.01 (Student t test). Underlying data can be found in S2 Data. 7-AAD, 7-amino-actinomycin D; BMP, Bone morphogenetic protein; BrdU, 5-bromo-2′-deoxyuridine; E8, Essential 8; FGF, Fibroblast growth factor; FUCCI, fluorescent ubiquitination–based cell-cycle indicator; hESC, human embryonic stem cell; ME, mesendoderm; NE, neuroectoderm; qPCR, quantitative PCR. (TIF) [file pbio.3000453.s002.tif]

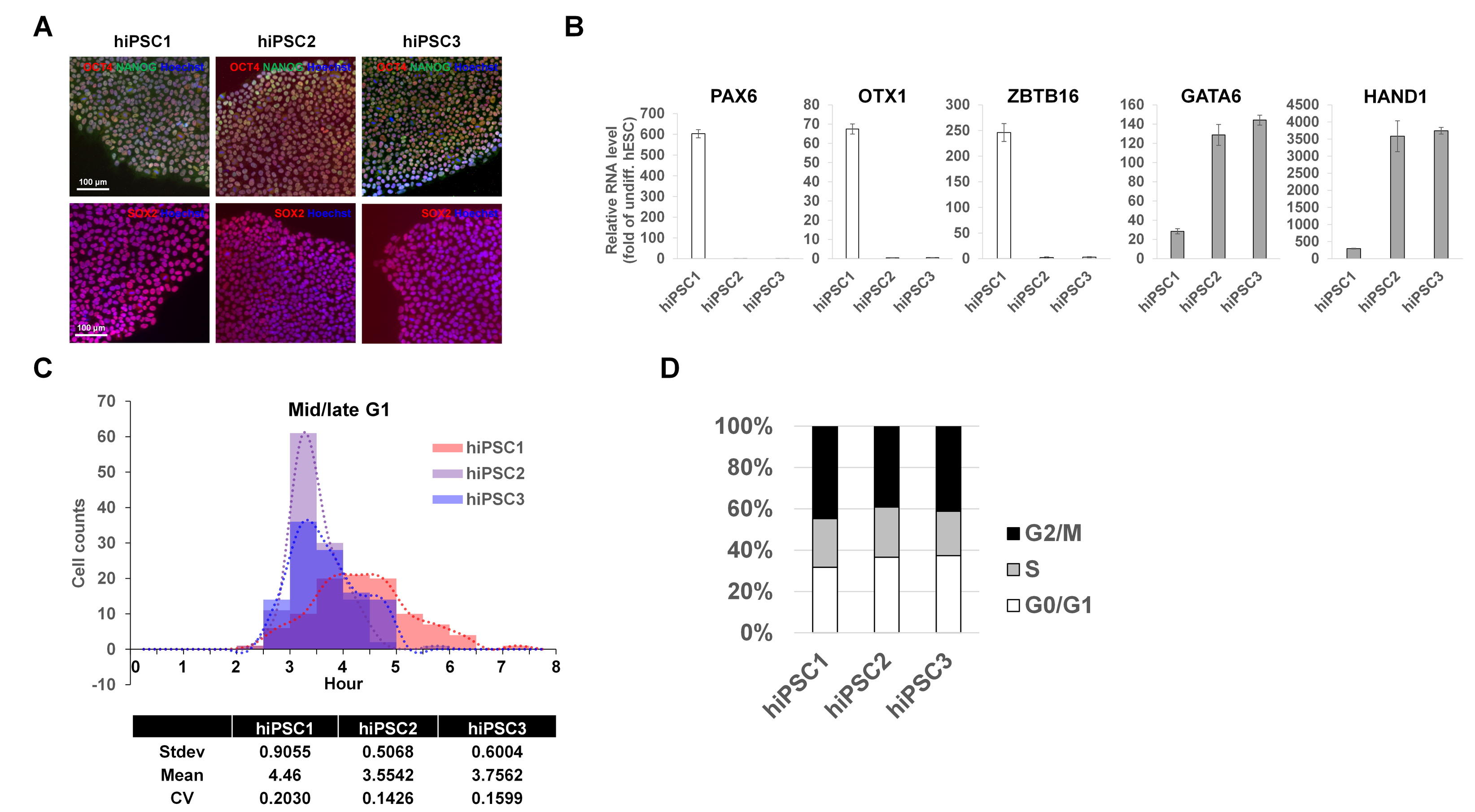

Supplement: S3 Fig — (A) Immunofluorescence of pluripotency genes in hiPSC lines grown in E8 medium. Representative images were shown from three independent experiments. (B) qPCR analysis of lineage markers in hiPSC lines differentiated for 7 d by FGF2 deprivation (n = 4). (C) Histograms for G1 length of hiPSC lines (n = 100 for hiPSC1, n = 120 for hiPSC2, and n = 108 for hiPSC3 pooled from two to three independent experiments); U test: p-value = 2.242 × 10^−14^ for hiPSC1 versus hiPSC2, p-value = 3.395 × 10^−9^ for hiPSC1 versus hiPSC3; KS test: p-value = 3.064 × 10^−14^ for hiPSC1 versus 2, p-value = 3.209 × 10^−7^ for hiPSC1 versus 3. (D) Propidium iodide staining analysis of hiPSC lines (n = 3). Error bars represent SD. *p < 0.01 (Student t test). Underlying data can be found in S2 Data. E8, Essential 8; FGF, Fibroblast growth factor; hiPSC, human induced pluripotent stem cell; KS, Kolmogorov-Smirnov; qPCR, quantitative PCR. (TIF) [file pbio.3000453.s003.tif]

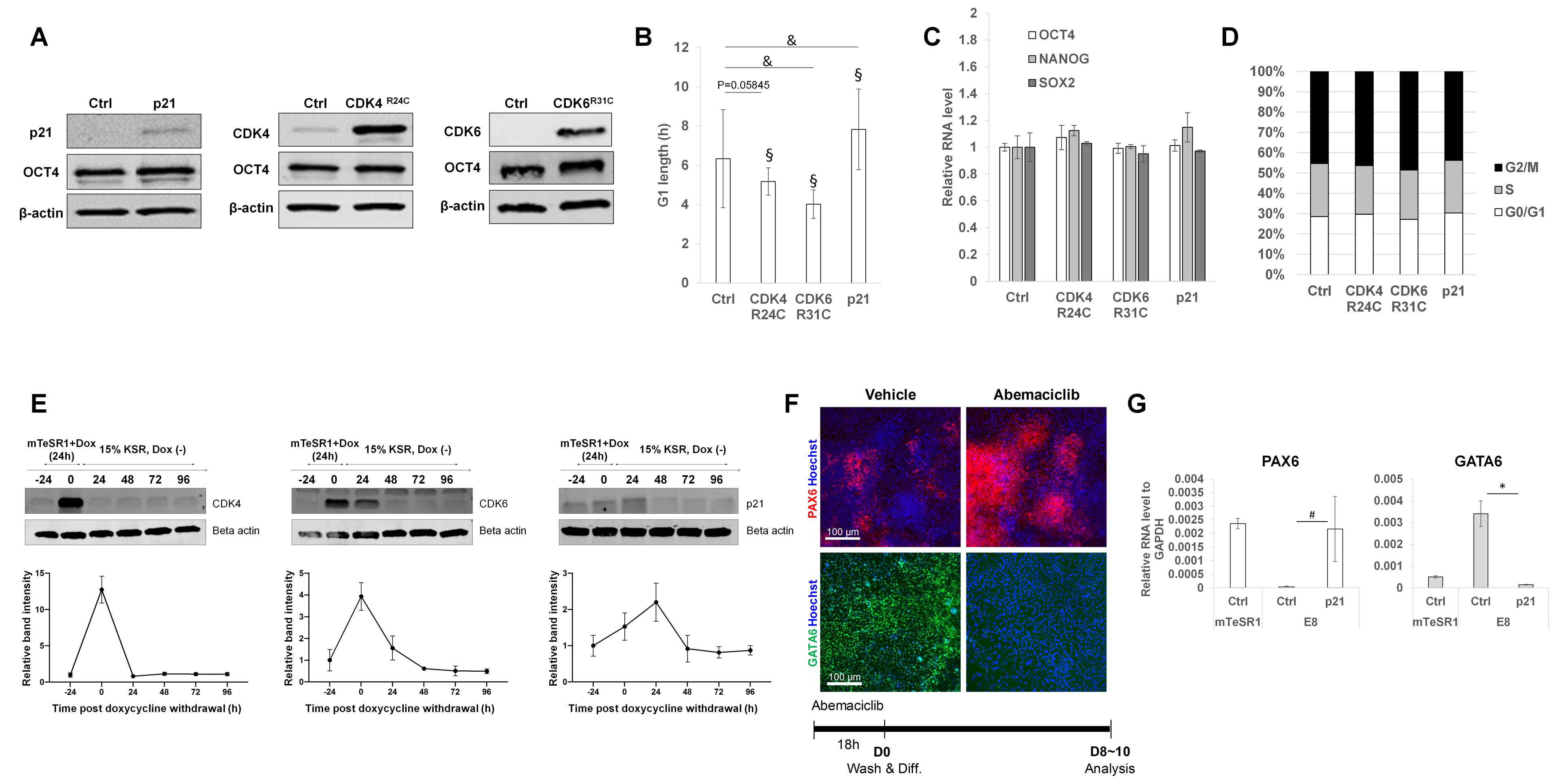

Supplement: S4 Fig — (A) Western blot of p21, CDK4, CDK6, and OCT4 in H9 cells expressing p21, CDK4R24C, or CDK6R31C. Representative images were shown from two independent experiments. (B) Average G1 length of FUCCI H9 cells expressing p21, CDK4R24C, or CDK6R31C (n = 54 for ctrl, n = 43 for p21, n = 52 for CDK4R24C, and n = 54 for CDK6R31C pooled from two independent experiments), &p < 0.01 (U test); §p < 0.01 (KS test). (C) qPCR analysis of pluripotency genes in H9 cells expressing p21, CDK4R24C, or CDK6R31C (n = 4). (D) Propidium iodide staining analysis of H9 cells expressing p21, CDK4R24C, or CDK6R31C (n = 3). (E) Dox-induced transgene expression and shutdown after Dox withdrawal in H9 cells transduced with p21, CDK4R24C, or CDK6R31C lentiviral vectors. Relative protein levels were analyzed by western blot assay (n = 3). (F) Immunofluorescence assay for PAX6 and GATA6 in H9 cells treated with Abemaciclib (0.5 μM) or vehicle (DMSO) for 18 h and then differentiated for 8–10 d. Representative images were shown from two independent experiments. (G) qPCR analysis of lineage markers in differentiated day 8 H9 cells overexpressing p21 (n = 4). Transgene expression was turned off at the onset of differentiation by Dox withdrawal. Error bars represent SD. #p < 0.05, *p < 0.01 (Student t test). Underlying data can be found in S2 Data. CDK, Cyclin-dependent kinase; ctrl, control; Dox, doxycycline; FUCCI, fluorescent ubiquitination–based cell-cycle indicator; GATA6, GATA binding protein 6; KS, Kolmogorov-Smirnov; OCT4, Octamer-binding transcription factor 4; PAX6, Paired box 6; qPCR, quantitative PCR. (TIF) [file pbio.3000453.s004.tif]

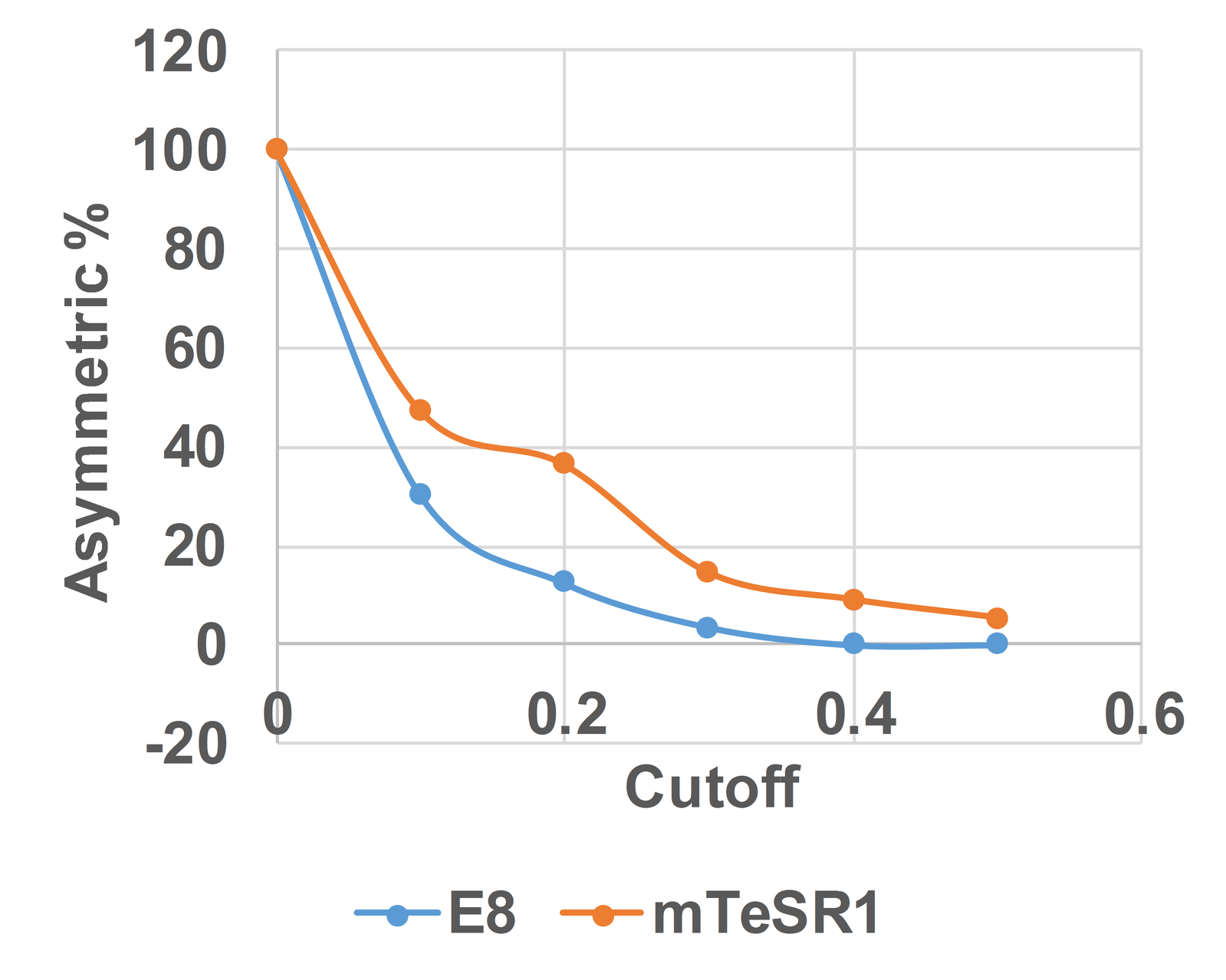

Supplement: S5 Fig — Difference in G1 length between sister cells (ΔG1) was divided by mean G1 length () between sister cells. Asymmetric sister cell G1 duration was defined by ΔG1/ values with various cutoffs (n = 56 for E8 and n = 55 for mTeSR1 pooled from three independent experiments). Underlying data can be found in S2 Data. E8, Essential 8. (TIF) [file pbio.3000453.s005.tif]

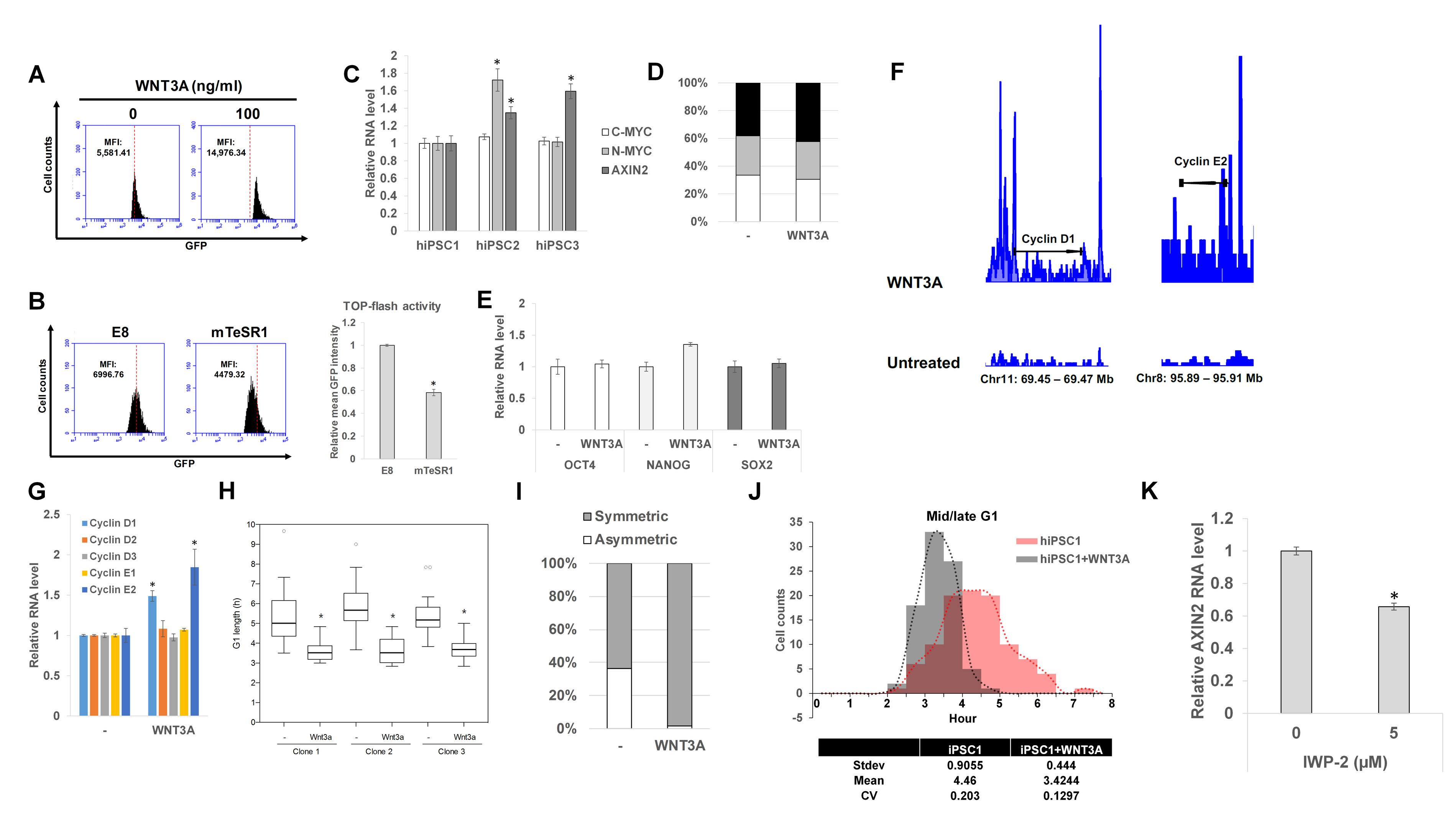

Supplement: S6 Fig — (A) Validation of TOP-flash reporter by recombinant WNT3A treatment. Representative images were shown from three independent experiments. (B) TOP-flash activity in H9 cells grown either in E8 or in mTeSR1 (n = 3). (C) qPCR analysis of WNT target genes in hiPSC lines grown in E8 (n = 4). (D) Propidium iodide staining analysis of H9 cells in mTeSR1 and treated with recombinant human WNT3A proteins (100 ng/ml) (n = 3). (E) qPCR analysis of pluripotency genes in H9 cells grown in mTeSR1 and treated with recombinant human WNT3A proteins (100 ng/ml) (n = 3~4). (F) Analysis of ChIP-seq peaks on the genomic loci of cyclins D1 and E2 with or without WNT3A treatment (GSE64758). Arrows represent genes. (G) qPCR analysis of cyclins D and E in H9 cells grown in mTeSR1 and treated with recombinant human WNT3A proteins (100 ng/ml) (n = 4). (H) G1 length of three clonal FUCCI lines treated with recombinant WNT3A (100 ng/ml) (n = 30 for each sample). *U test p-value < 0.0001. (I) Ratio of symmetric and asymmetric sister cell G1 durations in H9 cells grown in mTeSR1 and treated with recombinant human WNT3A proteins (100 ng/ml) (cutoff: ΔG1/ = 0.2, n = 55 for untreated and n = 52 for WNT3A pooled from three independent experiments). (J) Histograms for G1 length of hiPSC1 grown in E8 and treated with recombinant human WNT3A proteins (100 ng/ml) (n = 100 for hiPSC1 pooled from three independent experiments and n = 86 for hiPSC1 + WNT3A pooled from two independent experiments); U test: p-value < 2.2 × 10^−16^, KS test: p-value < 2.2 × 10^−16^. (K) qPCR analysis for AXIN2 expression in H9 cells treated with 5 μM IWP-2 (n = 4). Error bars represent SD. *p < 0.01 (Student t test). Underlying data can be found in S2 Data. ChIP-seq, chromatin immunoprecipitation followed by sequencing; E8, Essential 8; FUCCI, fluorescent ubiquitination–based cell-cycle indicator; hiPSC, human induced pluripotent stem cell; KS, Kolmogorov-Smirnov; MFI, mean fluorescence intensity; qPCR, quantitative PCR; WNT, [file pbio.3000453.s006.tif]

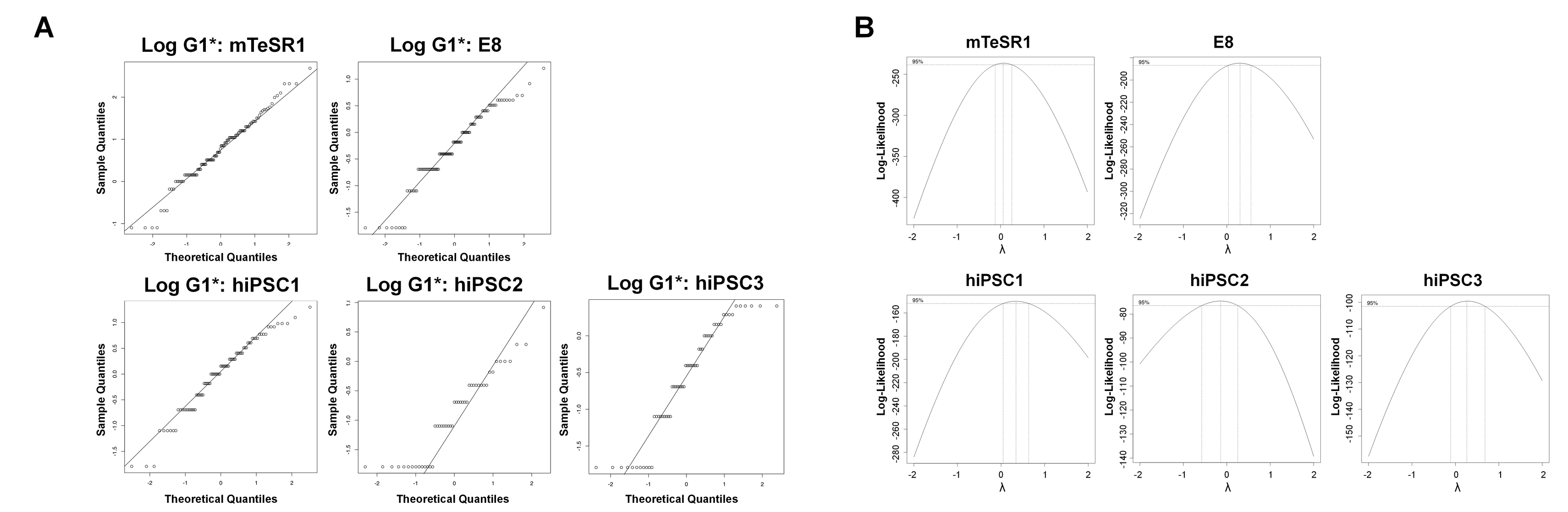

Supplement: S7 Fig — (A) q-q plots comparing the quantiles of log(G1*) with the quantiles of a normal distribution. (B) Box-Cox transformation of G1*. Underlying data can be found in S2 Data. (TIF) [file pbio.3000453.s007.tif]

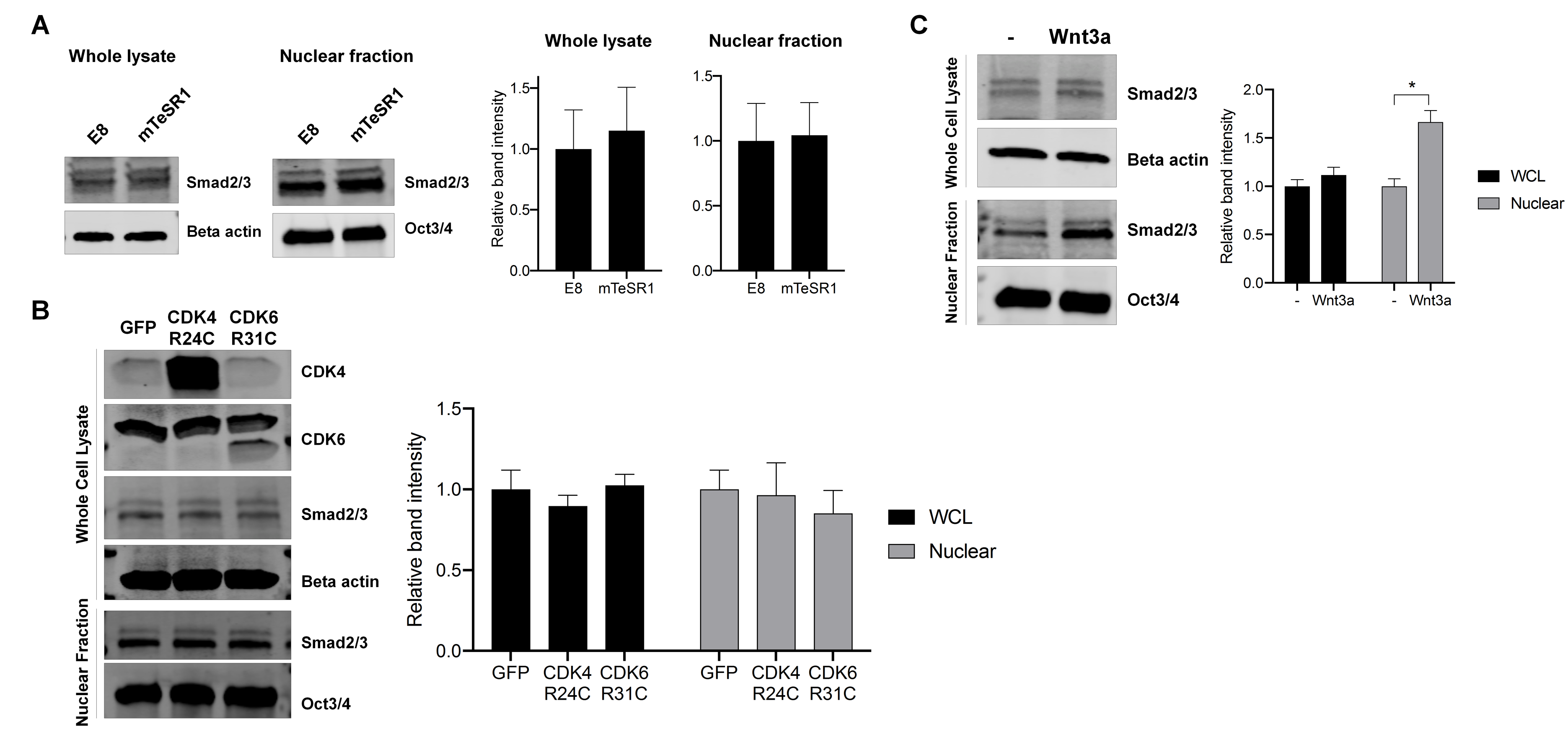

Supplement: S8 Fig — (A) Western blot of SMAD2/3 in nuclear and total fractions of H9 cells grown either in E8 or in mTeSR1 (n = 4). (B) Western blot of SMAD2/3 in nuclear and total fractions of H9 cells overexpressing CDK4R24C or CDK6R31C (n = 3). (C) Western blot of SMAD2/3 in nuclear and total fractions of H9 cells treated with 100 ng/ml of WNT3A for 24 h (n = 3). Error bars represent SD. *p < 0.01 (Student t test). Underlying data can be found in S2 Data. CDK, Cyclin-dependent kinase; E8, Essential 8; hESC, human embryonic stem cell; WNT, Wingless-INT. (TIF) [file pbio.3000453.s008.tif]

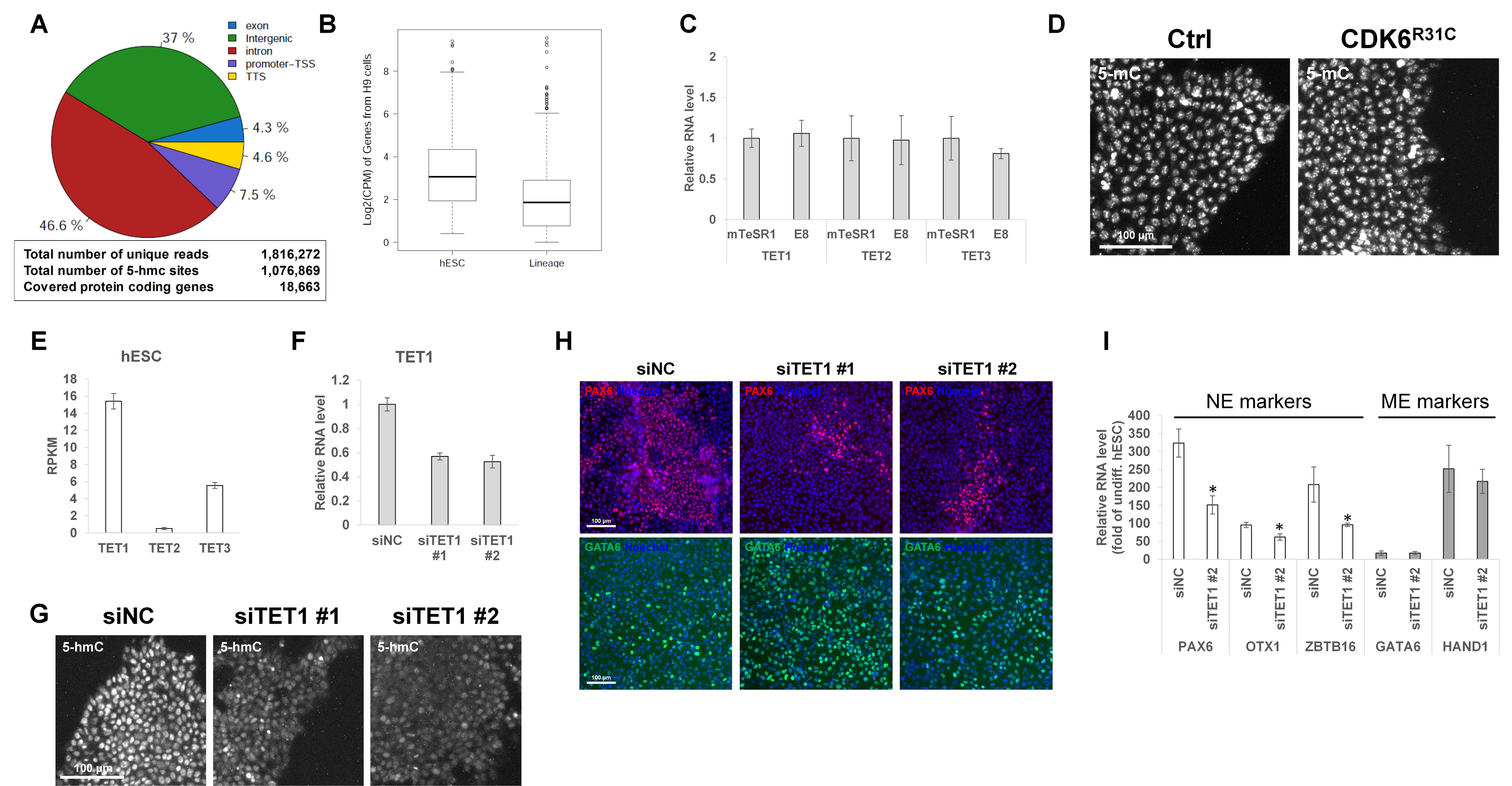

Supplement: S9 Fig — (A) Pie plot for annotated genome categories of 5-hmC sites detected in H9 hESCs grown in mTeSR1. (B) Gene expression patterns of hESC-specific and lineage-specific genes in H9 hESCs (U test: p-value < 2.2 × 10^−16^) (GSE69982). (C) qPCR analysis of TET1, TET2, and TET3 in H9 cells grown either in E8 or mTeSR1 (n = 4). (D) Immunofluorescence assay for 5-mC in H9 cells grown in mTeSR1 expressing CDK6R31C. Representative images were shown from three independent experiments. (E) Gene expression levels of TET1, TET2, and TET3 in hESCs (GSE69982). (F) qPCR analysis of TET1 in H9 cells transfected with siRNAs (n = 3). (G) Immunofluorescence assay for 5-hmC in H9 cells transfected with siRNAs. Representative images were shown from three independent experiments. (H) Immunofluorescence assay for PAX6 and GATA6 in H9 cells transfected with siRNAs and then differentiated for 9 d. Representative images were shown from three independent experiments. (I) qPCR analysis of lineage markers in H9 cells grown in mTeSR1, transfected with TET1 siRNA #2, and then differentiated for 8 d without FGF2 (n = 4). Error bars represent SD. *p < 0.01 (Student t test). Underlying data can be found in S2 Data. 5-hmC, 5-hydroxymethylcytosine; 5-mC, 5-methylctosine; CDK, Cyclin-dependent kinase; E8, Essential 8; FGF, Fibroblast growth factor; GATA6, GATA binding protein 6; hESC, human embryonic stem cell; PAX6, Paired box 6; qPCR, quantitative PCR; siRNA, small interfering RNA; TET, ten-eleven translocation. (TIF) [file pbio.3000453.s009.tif]
