# Supplementary material for: Control over single-cell distribution of G1 lengths by WNT governs pluripotency
Source: PLoS Biol. 2019 Sep 26;17(9):e3000453. doi: 10.1371/journal.pbio.3000453 (PMC6782112; doi:10.1371/journal.pbio.3000453)
Supplement: S1 Table — qPCR, quantitative PCR. (DOCX) [file pbio.3000453.s010.docx]

**S1 Table. Q-PCR primers used in this study.**

| Gene | Forward | Reverse |
| --- | --- | --- |
| OCT4 | GAGAAGGAGAAGCTGGAGCA | AATAGAACCCCCAGGGTGAG |
| NANOG | GATTTGTGGGCCTGAAGAAA | CAGATCCATGGAGGAAGGAA |
| SOX2 | CATGGACAGTTACGCGCACAT | AGTTGTACTGCAGGGCGCTCA |
| PAX6 | GGCTAGCGAAAAGCAACAGA | TGGTATTCTCTCCCCCTCCT |
| OTX1 | CACCGCAGCAGCCTCTTATCC | TGCATGGGCGCTAGGTATGAG |
| ZBTB16 | AAGCGGTTCCTGGATAGTTTG | CATGTCAGTGCCAGTATGGGT |
| GATA6 | AGGGCTCGGTGAGTCCAAT | CGCTGCTGGTGAATAAAAAGGA |
| HAND1 | TCAGCCTTGCCCGGACTCTC | AGGTTCATGTTGGAGCGGCTAC |
| Brachyury | ACAATGCCAGCCCACCTACCAG | CGTACTGGCTGTCCACGATGTCTG |
| SOX17 | CGCTTTCATGGTGTGGGCTAAG | CGCTCTGCCTCCTCCACGAAG |
| EOMES | TGTTCGTAGAGGTGGTGCTG | ATTTGCGCCTTTGTTATTGG |
| CCND1 | CAAACAGATCATCCGCAAACA | ACTCTGGAGAGGAAGCGTGTG |
| CCND2 | CTCTGTGTGCCACCGACTTTA | TGGCAAGCTTTGAGACAATCC |
| CCND3 | CTTCATTCTGCACCGGCTCT | GATCATGGATGGCGGGTACAT |
| CCNE1 | CGGTATATGGCGACACA | CCATCTGTCACATACGCAAAC |
| CCNE2 | CCAAGAAGCCCAGATAATCC | AGGTGGCCAACAATTCCTAAT |
| CCNA1 | GGGCTCCCAGATTTCGTCTTC | TGACAAGCATCGGGACCTC |
| CCNA2 | AAGACGAGACGGGTTGCAC | CAGGGCATCTTCACGCTCTAT |
| CCNB1 | AAGGGAGTGAGTGCCACGAA | GACCAGCCAAGGACCTACACC |
| CCNB2 | TCAATTAGTTGGGATTACTGC | GATTTGGGAACTGGTATAAGC |
| CCNB3 | TGCCACTGCCTTTATGATTGC | GGCGATGGGAATGTTAATGTC |
| C-MYC | CCCTCCTACGTTGCGGTCACA | CGTCCGGGTCGCAGATG |
| N-MYC | GGAAGAAATCGACGTGGTCAC | GGTTTCTGCGACGCTCACT |
| AXIN2 | ACCACCATTCGCAGTACCACT | GACATGCTTCGTCGTCTGCT |
| TET1 | TTTCACAGCTTGGCGAAGTG | GTTTGGCTGATGAGGCGTTAG |
| GAPDH | GAAGGTGAAGGTCGGAGTC | GAAGATGGTGATGGGATTTC |
